# Standard vs. carbone dioxide adapted kidney replacement therapy in hypercapnic ARDS patients: a randomized controlled pilot trial (BigBIC)

**DOI:** 10.1186/s13054-024-04979-z

**Published:** 2024-06-11

**Authors:** Julius Valentin Kunz, Helena Hansmann, Mareike Fähndrich, Mareen Pigorsch, Nicole Bethke, Harm Peters, Anne Krüger, Tim Schroeder, Florian Marcy, Abakar Magomedov, Holger Müller-Redetzky, Kai-Uwe Eckardt, Dmytro Khadzhynov, Philipp Enghard

**Affiliations:** 1 Department of Nephrology and Medical Intensive Care, Charité – Universitätsmedizin Berlin, Corporate Member of Freie Universität Berlin, Humboldt Universität zu Berlin and Berlin Institute of Health, Augustenburger Platz 1, 13353 Berlin, Germany; 2 Present Address: Department of Nephrology, University Medical Center Regensburg, Franz-Josef-Strauss-Allee 11, 93053 Regensburg, Germany; 3 Institute of Biometry and Clinical Epidemiology, Charité – Universitätsmedizin Berlin, Corporate Member of Freie Universität Berlin, Humboldt Universität zu Berlin and Berlin Institute of Health, Charité-Platz 1, 10117 Berlin, Germany; 4 Present Address: Department of Nephrology, Vivantes Klinikum Friedrichshain, Berlin, Germany; 5 Department of Infectious Diseases, Pneumology and Intensive Care Medicine, Charité – Universitätsmedizin Berlin, Corporate Member of Freie Universität Berlin, Humboldt Universität zu Berlin and Berlin Institute of Health, Charité-Platz 1, 10117 Berlin, Germany; 6 Present Address: Kuratorium for Dialysis and Transplantation (KfH) Renal Unit Berlin-Mitte, Große Hamburger Str. 5-11, Berlin, Germany

**Keywords:** Acute respiratory distress syndrome, Hypercapnia, Kidney replacement therapy, Mechanical ventilation, Respiratory acidosis

## Abstract

**Background:**

Current continuous kidney replacement therapy (CKRT) protocols ignore physiological renal compensation for hypercapnia. This study aimed to explore feasibility, safety, and clinical benefits of pCO2-adapted CKRT for hypercapnic acute respiratory distress syndrome (ARDS) patients with indication for CKRT.

**Methods:**

We enrolled mechanically ventilated hypercapnic ARDS patients (pCO2 > 7.33 kPa) receiving regional citrate anticoagulation (RCA) based CKRT in a prospective, randomized-controlled pilot-study across five intensive care units at the Charité—Universitätsmedizin Berlin, Germany. Patients were randomly assigned 1:1 to the control group with bicarbonate targeted to 24 mmol/l or pCO_2_-adapted-CKRT with target bicarbonate corresponding to physiological renal compensation. Study duration was six days. Primary outcome was bicarbonate after 72 h. Secondary endpoints included safety and clinical endpoints. Endpoints were assessed in all patients receiving treatment.

**Results:**

From September 2021 to May 2023 40 patients (80% male) were enrolled. 19 patients were randomized to the control group, 21 patients were randomized to pCO_2_-adapted-CKRT. Five patients were excluded before receiving treatment: three in the control group (consent withdrawal, lack of inclusion criteria fulfillment (n = 2)) and two in the intervention group (lack of inclusion criteria fulfillment, sudden unexpected death) and were therefore not included in the analysis. Median plasma bicarbonate 72 h after randomization was significantly higher in the intervention group (30.70 mmol/l (IQR 29.48; 31.93)) than in the control group (26.40 mmol/l (IQR 25.63; 26.88); *p* < 0.0001). More patients in the intervention group received lung protective ventilation defined as tidal volume < 8 ml/kg predicted body weight. Thirty-day mortality was 10/16 (63%) in the control group vs. 8/19 (42%) in the intervention group (*p* = 0.26).

**Conclusion:**

Tailoring CKRT to physiological renal compensation of respiratory acidosis appears feasible and safe with the potential to improve patient care in hypercapnic ARDS.

**Trial registration:**

The trial was registered in the German Clinical Trials Register (DRKS00026177) on September 9, 2021 and is now closed.

**Supplementary Information:**

The online version contains supplementary material available at 10.1186/s13054-024-04979-z.

## Background

Lung protective ventilation with low tidal volumes (< 8 ml/kg predicted body weight (PBW)) and low inspiratory pressures (plateau pressure < 30 cmH2O) is the centerpiece of standard therapy in patients with Acute Respiratory Distress Syndrome (ARDS) according to international guidelines [1, 2]. In low tidal ventilation moderate increase of carbon dioxide partial pressure (pCO_2_) is tolerated in favor of lung protection (permissive hypercapnia). In individuals with normal kidney function pCO_2_ retention is compensated via increased bicarbonate (HCO_3-_) reabsorption and net acid excretion in the kidney to counterbalance acidosis [3]. Adequate compensation in chronic respiratory acidosis is equivalent to a factor of 3 (HCO_3-_ [mmol/L] increase per CO_2_ [kPa] increase) [4]. Acute kidney injury (AKI) is a common complication in ARDS patients and affects approximately two thirds of patients [5, 6]. In patients with chronic kidney disease (CKD) and/or AKI, the ability to balance the acid–base budget is impaired. In patients with severely impaired kidney function undergoing continuous kidney replacement therapy (CKRT), the ability to metabolically compensate a pCO_2_ increase is almost completely lost. This implies a more acidic pH in cases of pCO_2_ retention, which limits the tolerable pCO_2_ level for lung-protective ventilation. Moreover, acidosis may have additional detrimental effects of its own. To avoid a pronounced pH shift (pH < 7.2), buffer substances (e.g., sodium bicarbonate, trometamol (TRIS)) can be used. However, there is therapeutic uncertainty about their use and buffer substances are not free of possible side-effects such as volume overload, hyperosmolarity and a possible additional CO_2_ load [3]. In CKRT with regional citrate anticoagulation (RCA), certain HCO_3-_ levels can be achieved by adjusting the blood-to-dialysate ratio regardless of chosen CKRT-modality [7, 8]. As common dialysate solution for RCA usually has reduced bicarbonate concentration to account for buffering capacities of citrate, RCA-CKRT provides a perfect setting to reach a tailored metabolic state, allowing treatment for both, metabolic acidosis and alkalosis [8, 9]. Changing the blood-to-dialysate ratio leads to an in- or decrease in citrate dose delivered to the patients, where citrate is subsequently metabolized, leading to concordant changes of circulating HCO_3-_ levels. Therefore, RCA-CKRT can mimic metabolic compensation of respiratory acidosis and substitute the physiologic kidney function in this regard. Using such a CKRT-based metabolic compensation of elevated pCO_2_ levels is not part of routine clinical care and we are not aware of any studies that have investigated this approach. A pCO_2_-adapted bicarbonate target in CKRT could avoid possible adverse effects of intravenous alkali therapy via slow and continuous regulation of acid–base balance while controlling fluid balance [3]. By changing the blood-to-dialysate ratio according to the expected physiological renal compensation, in ARDS patients undergoing CKRT, lung-protective ventilation could be facilitated or enabled, avoiding acidosis as limiting factor.

Aim of this prospective randomized-controlled trial is to investigate the feasibility and safety of a pCO_2_-adapted bicarbonate target in CKRT and evaluate potential clinical benefits on ventilation in hypercapnic ARDS patients.

## Material and methods

### Study design

The BigBIC-study is a prospective, single-center, open-label, randomized controlled pilot study to investigate the feasibility, safety and clinical benefits of a pCO_2_-adapted RCA-CKRT in hypercapnic ARDS patients requiring kidney replacement therapy. The study was conducted on five intensive care units (ICUs) at three sites of the university hospital Charité in Berlin, Germany. The study was approved by the local ethics committee (EA2/101/21). The study protocol can be found in the supplementary material. The trial was registered in the German Clinical Trials Register (DRKS00026177) on September 9, 2021 and is now closed. The trial was conducted according to the Declaration of Helsinki.

### Patient selection

All patients aged ≥ 18 years with invasive mechanical ventilation due to ARDS of any cause defined by the Berlin Definition [10], without time restriction and indication for CKRT performed with regional citrate anticoagulation were eligible for study inclusion. Patients had to be hypercapnic defined as pCO_2_ > 7,33 kpa with tidal volumes > 4 ml/kg and respiratory rate ≥ 12/min. Patients on extracorporeal membrane oxygenation (ECMO), patients receiving TRIS-buffering and patients with lactate acidosis (lactate > 80 mg/dl) or liver failure, defined as bilirubin > 8 mg/dl and INR > 2 were excluded. Written informed consent was obtained from patients or their legal representatives. For patients incapable of giving consent and without a legal representative, urgent appointment of a legal guardian was requested. At inclusion, the attending physician was asked to confirm patient eligibility.

### Randomization and masking

A 1:1 randomization was performed by pulling sealed envelopes allocating patients to either the control group or the intervention treatment. The investigators performed the consecutive patient screening and enrolment. As blinding was not feasible, therapy was applied in an open-label fashion.

### Procedures

Outside the trial intervention all patients received standard intensive care treatment according to ARDS guidelines [1, 2], including aiming for lung protective ventilation with low tidal volumes (4–8 ml/kgPBW) and limited inspiratory pressures (inspiratory plateau pressure (IPP) < 30 cmH2O).

Until study inclusion RCA-CKRT was provided according to a previously published protocol [8] aiming for a therapy dose of 20–25 ml/kg/h. Briefly, RCA-CKRT was conducted using high-flux dialysers (AV1000, Fresenius Medical Care (FMC), Bad Homburg, Germany), 4% trisodium citrate solution (136 mmol/L; Fresenius Kabi, Bad Homburg, Germany) and dialysate solution (CiCa K2, Fresenius Medical Care (FMC), Bad Homburg, Germany).

After randomization, patients received either conventional care with bicarbonate targeted to 24 mmol/L or the study intervention with bicarbonate targeted to ((pCO_2_-6 kPa)/1,33 kPa)*4 mmol/l + 24 mmol/l according to the physiological renal compensation. Buffering via RCA-CRKT is achieved via administration of citrate, which is concomitantly metabolized to bicarbonate. To avoid a metabolic acidosis due to the additional citrate administration, the RCA-CKRT dialysis solution which was used contains less bicarbonate than other dialysate solutions (20 mmol/l bicarbonate). The dialysis machines which were used (multiFiltrate CiCa Fresenius Medical Care (FMC), Bad Homburg, Germany) are equipped with an automated control system that regulates citrate dosing: Using this system a constant concentration of citrate in the blood of 4 mmol/l citrate/L blood was maintained. As a result, the amount of citrate administered increases if the blood-flow is increased without a corresponding increase in dialysate-flow. Raise of bicarbonate concentration in RCA-CKRT can therefore be achieved via either increasing the blood-flow and thus concomitantly the citrate dose which is metabolized to bicarbonate or reducing the dialysate-flow (as less bicarbonate and citrate is cleared from the patients´ blood). In this trial in the intervention group the blood-dialysate flow adjustment was standardized: in a first step the blood flow was raised by 20 ml/min (which corresponds to an additional citrate flow of 4,8 mmol/h). If HCO_3_- was below the target after 12 h the dialysate-flow was then reduced by 200 ml/hour. More details on further adjustments are provided in Supplemental Fig. 1 and Supplemental Table 1. The intervention was intended to span six days, involving a filter exchange every 72 h.

**Fig. 1:**
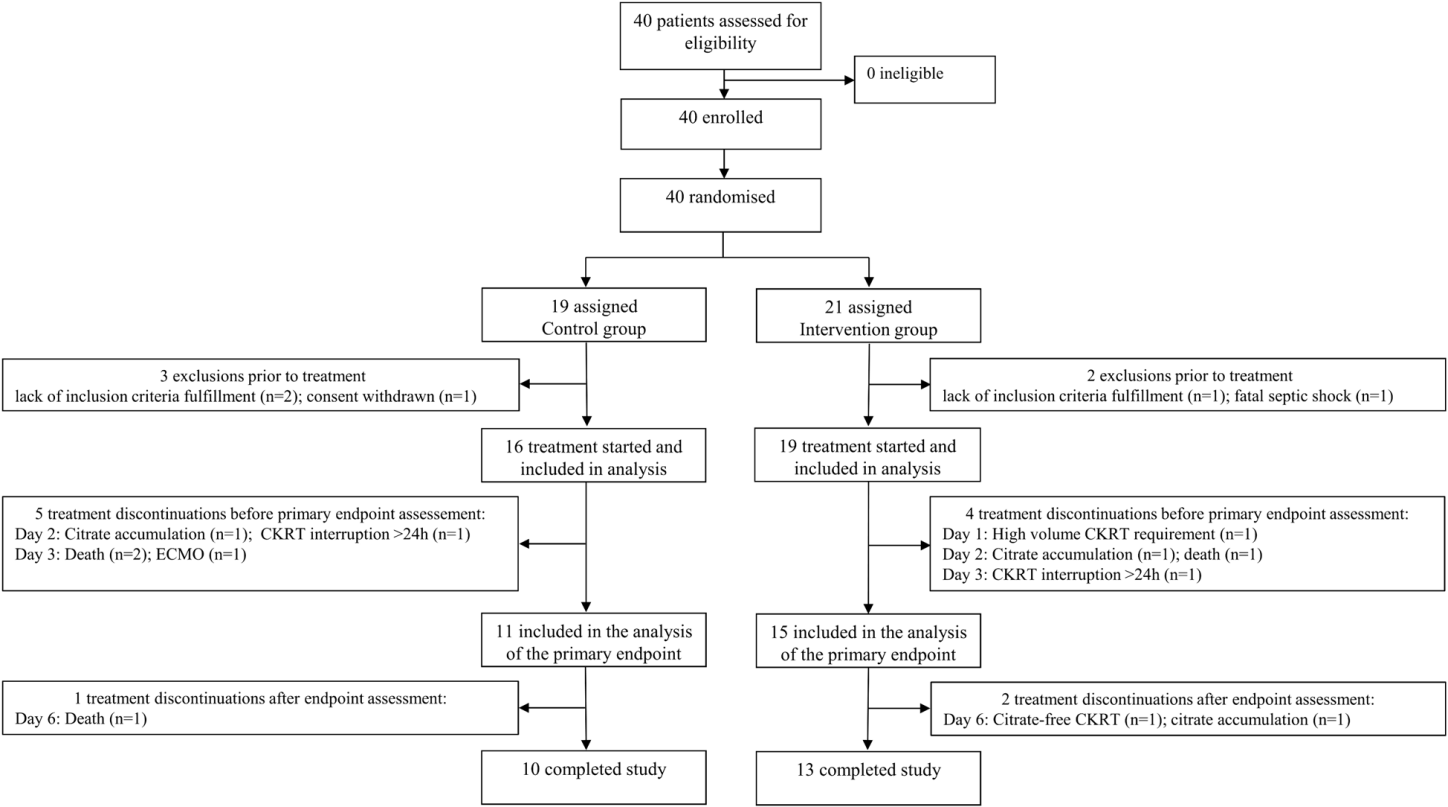
**Patient disposition. **Endpoints were assessed in all patients in whom treatment was started. Endpoints were analyzed as long as treatment was applied until its discontinuation. Mortality data was recorded until ICU discharge for all patients in whom treatment was started. The reason for study discontinuation was the first reason which occurred. Multiple reasons for treatment discontinuation were possible

**Table 1:** Baseline characteristics of the study cohort

	N	Control group	Intervention group
		N = 16	N = 19
Age, years	35	65 (59, 73)	60 (50, 68)
Female*	35	3 (19%)	4 (21%)
BMI, kg/m^2^	35	25.5 (23.3, 27.7)	27.8 (25.6, 34.9)
Predicted body weight, kg	35	67 (59, 73)	75 (65, 80)
*Comorbidities*			
Chronic kidney disease (stage II-IV)	35	7 (44%)	4 (21%)
Hypertension	35	10 (63%)	9 (47%)
Diabetes	35	3 (19%)	3 (16%)
Coronary artery disease	35	6 (38%)	7 (37%)
Chronic obstructive pulmonary disease	35	3 (19%)	3 (16%)
History of malignancy	35	6 (38%)	7 (37%)
Immunosuppressive therapy	35	9 (56%)	4 (21%)
*Clinical characteristics at study inclusion*			
SOFA Score (ICU admission)	35	10.0 (7.5, 13.0)	10.0 (8.5, 13.5)
SOFA (study inclusion)	35	11.50 (10.75, 14.25)	13.00 (12.00, 14.00)
PaO_2_/FiO_2_, mmHg	35	168 (126, 229)	146 (118, 191)
Norepinephrine dose, µg/kg/min	35	0.19 (0.10, 0.34)	0.14 (0.09, 0.19)
Other vasopressors	35	1 (6.3%)	1 (5.3%)
COVID-19	35	8 (50%)	9 (47%)
*Acid–base parameters at study inclusion*			
pH	35	7.28 (7.23, 7.31)	7.26 (7.21, 7.29)
HCO_3_, mmol/L	35	27.85 (25.13, 28.43)	26.00 (25.35, 27.70)
pCO_2_, kPa	35	7.95 (7.65,8.45)	8.08 (7.89, 8.63)
*Ventilatory parameters at study inclusion*			
Tidal volume/kg PBW, ml	34	6.00 (5.50, 7.97)	6.17 (5.57, 7.59)
Tidal volume < 8 ml/kg PBW	34	11 (73%)	16 (84%)
Positive end-expiratory pressure, mBar	33	13.0 (11.0, 15.0)	15.0 (14.0, 18.0)
Driving pressure, mBar	34	15.00 (11.50, 17.00)	13.00 (11.00, 14.50)
Inspiratory plateau pressure, mBar	33	27.0 (23.3, 29.0)	29.0 (26.0, 31.0)
Respiratory minute volume/kg PBW, mL	33	138 (128, 165)	152 (131, 186)
Respiratory rate, breaths/min	33	23 (21, 27)	23 (21, 26)
*Laboratory parameters at study inclusion*			
CRP, mg/dL	35	211 (53, 250)	165 (134, 266)
PCT, ug/L	35	2 (1,8)	3 (1,7)
ALT, U/L	35	76 (32, 187)	35 (20, 93)
AST, U/L	35	126 (58, 354)	67 (50, 262)
gGT, U/L	35	145 (68, 396)	141 (66, 312)
Bilirubine, mg/dl	35	0.70 (0.33, 1.21)	1.14 (0.53, 2.30)
Lactate, mg/dl	35	13.5 (8.8, 19.0)	13.0 (6.5, 18.0)
Sodium, mmol/L	35	143.50 (143.00, 145.05)	143.00 (142.00, 144.00)
Ionised serum calcium, mmol/l	35	1.20 (1.16, 1.24)	1.17 (1.14, 1.20)
Calciumdose, mmol/L	35	1.70 (1.65, 1.95)	1.70 (1.50, 1.90)

Values are medians (IQR) or N = number (%); ALT: alanine aminotransferase; AST: aspartate aminotransferase; BMI: body mass index; CRP: C-reactive protein; gGT: gamma GT; HCO3: hydrogen carbonate; PBW: predicted body weight; pCO2: Carbon dioxide partial pressure; SOFA: sequential organ failure assessment score

^*^biological sex (sex assigned at birth), information on gender was not collected

### Outcomes

Endpoints included efficacy endpoints, safety endpoints and clinical endpoints. The primary efficacy endpoint was the plasma bicarbonate concentration 72 h after study inclusion in all patients receiving the intervention for at least 72 h. Secondary efficacy endpoint was the kinetics of HCO_3-_ concentrations measured every 12 h in both groups. Secondary safety endpoints were predefined adverse events: mortality (mortality during the intervention period of six days, 30-day mortality and mortality until ICU-discharge), severe acidosis (defined as pH < 7.15), severe alkalosis (defined as pH > 7.55), severe hypernatremia (defined as plasma sodium > 155 mmol/l), severe hypo- or hypercalcemia (defined as ionized Ca_2+_  < 0.8 mmol/l or > 1.5 mmol/l), severe hypo- or hyperkalemia (defined as potassium < 2.5 mmol/l or > 6.5 mmol/l), severe hypophosphatemia (defined as plasma phosphate < 0.3 mmol/l), filterclotting and citrate accumulation in both groups during the time of intervention of six days. The switch to citrate-free CKRT due to suspected citrate accumulation was done at the discretion of the treating physicians. For outcome assessment the study team evaluated all cases of suspected citrate accumulation. Citrate accumulation was considered confirmed if at least three of four generally accepted systemic metabolic criteria were present: (1) decrease of systemic ionized calcium (iCa) (< 1.1 mmol/L); (2) concomitant increase of total calcium concentration and, thus, an increase of total to iCa ratio (> 2.25); (3) relevant metabolic acidosis (pH < 7.2 and/or base excess < –5 mmol/L); and (4) elevated anion gap (> 12 mmol/L) 11. Secondary clinical endpoints were the median of the following parameters over the intervention period of six days: pH, tidal volumes, driving pressure, peak pressure, respiratory minute volume, catecholamine dose, the duration of mechanical ventilation, sequential organ failure assessment (SOFA)-score and the daily blood flow and dialysate flow in RCA-CKRT. In cases where there was a change in CKRT modality (e.g. switching to RCA-free CKRT due to confirmed or suspected citrate accumulation), any adjustments to CKRT outside the study intervention, CKRT interruption exceeding 24 h, initiation of ECMO therapy, or TRIS-buffering the treatment was discontinued, as the evaluation of the intervention's effect was not feasible under these circumstances. In those patients, endpoints were analyzed as long as treatment was performed until treatment discontinuation. Mortality was recorded until ICU-discharge in all patients in whom treatment was started.

### Statistical analysis

The study was conducted as an investigator-initiated pilot study to explore the feasibility and safety of the intervention and the treatment effects.

Sample size calculation was done using the Wilcoxon–Mann–Whitney rank sum test for continuous outcomes and the nQuery version 8.7.2 program. In a previous randomized controlled trial [12] the median bicarbonate in 48 patients with normal liver function at dialysis initiation was 20.9 mmol/l ± 4.8. 72 h after dialysis initiation, the median bicarbonate was 25.2 mmol/l ± 2.4. In our study a median HCO_3-_ of 24 mmol/l was assumed in the control group and a median HCO_3_ of 32 mmol/L was assumed in the intervention group (corresponding to a paCO_2_ of 8,67 kPa according to the formula). In the intervention group a larger standard deviation was calculated than in the above-mentioned study because the patients have different target bicarbonate levels. Therefore, a common standard deviation of 8 was assumed. To demonstrate this effect with a power of 80% at a significance level of 5%, a sample size of 20 per group was calculated.

To compare metric outcomes between control and intervention group, the Brunner-Munzel test was used due to non-normally distributed values with heterogeneous variances. The test was performed using the function rank.two.samples() from the R package rankFD [13]. The effect measure of the Brunner-Munzel test is the relative effect *p* with the null hypothesis *p* = 1/2. The relative effect *p* is to be interpreted as follows: If p(control,intervention) > 1/2, the data in the intervention group tends to be larger than the data in control group; conversely, if *p* < 1/2, the data in the intervention group tends to be smaller than the data in the control group. The odds were calculated as p/(1-p). For comparison of relative frequencies, the Boschloo’s test was used due to small sample sizes. The test was performed using the function exact.test() from the R package Exact [14]. The significance level for the primary endpoint was set to 0.05. For all secondary endpoints, the analyses are explorative and do not allow for confirmatory conclusions. Analysis and graphical preparation of the data was performed with R Version −4.2.2 [15]. In the boxplots, outliers were determined using the IQR method, where data points beyond 1.5 times the IQR from the first and third quartiles were identified and marked.

The mortality rates depicted in the Kaplan–Meier curve are derived from the cumulative probability of survival over time, considering the observed events. The *p*-value provided is based on the Log-Rank test.

### Role of the funding source

There was no funding source for this study.

## Results

From September 19, 2021, to May 31, 2023, 40 patients were enrolled, of which 19 patients were randomized to the control group and 21 patients were randomized to receive pCO_2_-adapted CKRT. Overall, five patients had to be excluded before receiving treatment: three in the control group (consent withdrawal (n = 1); after randomization patient did not fulfill the inclusion criteria anymore (n = 2)) and two in the intervention group (after randomization patient did not fulfill the inclusion criteria anymore (n = 1); sudden unexpected death before treatment initiation (n = 1)) and were therefore not included in the analysis.

After randomization treatment was started in 16 patients receiving the control treatment and 19 patients receiving pCO_2_-adapted CKRT (Fig. 1). Table 1 shows the baseline characteristics of the patients analyzed. In both groups about 80% of patients were male. Median age was 65 years (IQR 59; 73) in the control group and 60 years (IQR 50; 68) in the intervention group, BMI was slightly higher in the intervention group. Comorbidities were relatively well balanced between both groups, except that chronic kidney disease and immunosuppressive therapy were slightly more common in the control group.

Clinical characteristics and acid–base parameters were similar in both groups. At study inclusion, median paO_2_/FiO_2_ was 168 mmHg (IQR 126; 229) in the control group and 146 mmHg (IQR 118; 191) in the intervention group, respectively. Median tidal volume was around 6 ml/kg predicted body weight (PBW) in both groups. Median HCO_3-_ was 27.85 mmol/l (IQR 25.13; 28.43) in the control and 26.00 mmol/l (IQR 25.35; 27.70) in the intervention group.

As depicted in Fig. 1, treatment was discontinued in a total of 12 patients (six in the control group and six in the intervention group). In the control group, the reasons were as follows: death (n = 3), ECMO therapy (n = 1), citrate accumulation with need for RCA-free CKRT and simultaneous TRIS buffering (n = 1) and CKRT interruption > 24 h (n = 1). In the intervention group, the reasons were transitioning to RCA-free CKRT due to suspected citrate accumulation (n = 1), CKRT interruption > 24 h (n = 1), citrate accumulation with need for RCA-free CKRT and simultaneous TRIS buffering (n = 2), death (n = 1), and the need for a high dialysis dose due to therapy-resistant hyperkalemia (n = 1). Endpoints were assessed for all patients in whom treatment was initiated and up to the point of discontinuation. Due to nine treatment discontinuations by day 3, there were 26 patients evaluated for the primary endpoint of bicarbonate concentration on day 3. 10 patients in the control group and 13 patients in the intervention group completed the study.

**Fig. 2:**
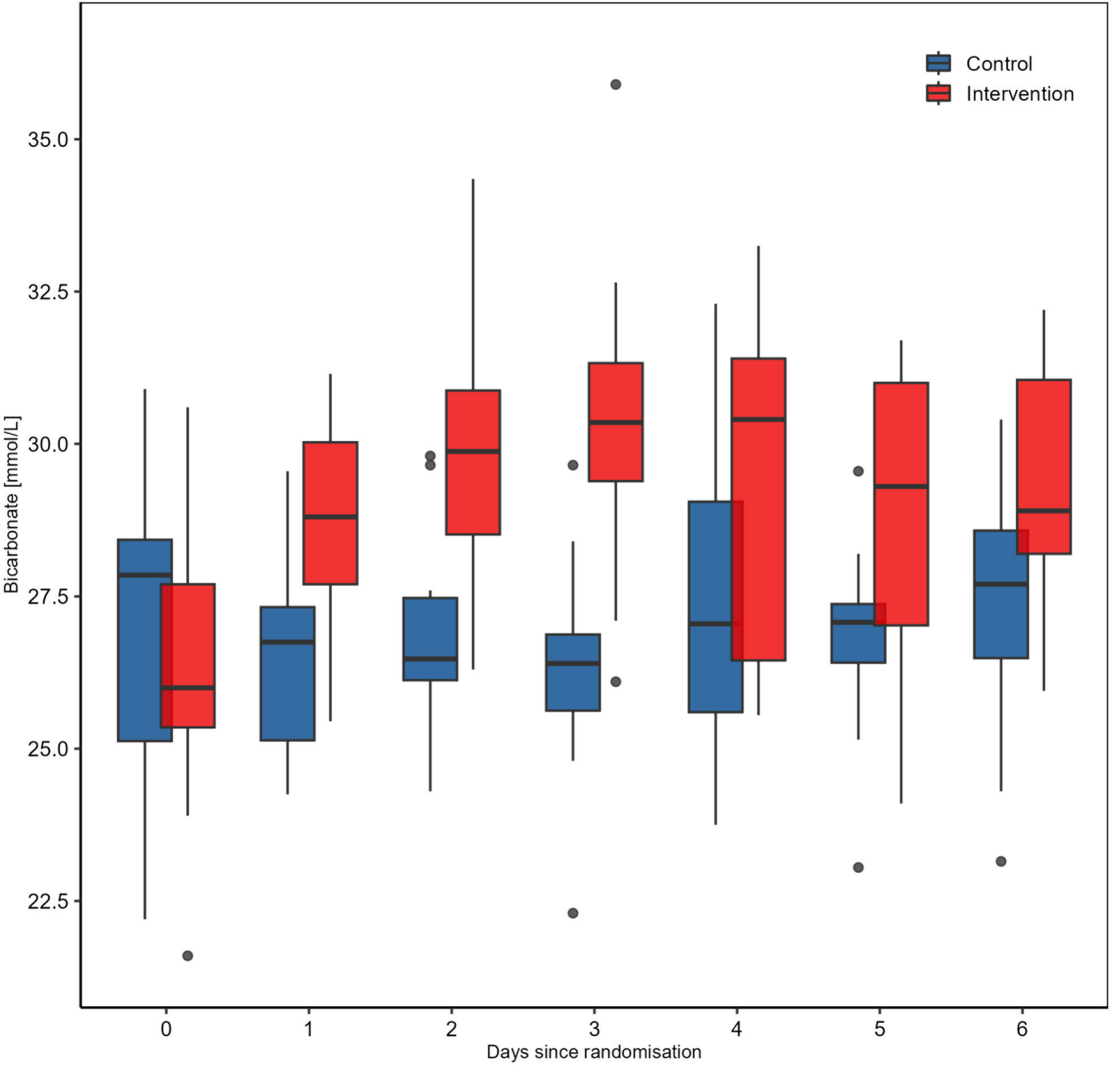
**Bicarbonate concentration over the study period. **Median bicarbonate concentration over time: The bicarbonate concentrations in the intervention group were consistently higher from day 1 to day 6 compared to the control group. Boxplots plotted over time. Outliers are displayed as dots (IQR method)

Figure 2 shows the plasma bicarbonate concentrations over time. The primary efficacy endpoint, the bicarbonate concentration 72 h after study inclusion, was significantly higher in the intervention group (30.70 mmol/l (IQR 29.48; 31.93)) as compared to the control group (26.40 mmol/l (IQR 25.63; 26.88)) (*p* < 0.0001). In fact, the bicarbonate concentration in the intervention group was higher from day one until day six with increasing difference until day 3 and decreasing difference afterwards (Table 2, Fig. 2).

**Table 2:** Outcomes of the study participants

	N^1^	Control group^2^	Intervention group^2^	Relative effect^3^ (CI)^4^	Odds^5^	*p*-value^6^
		N = 16	N = 19			
*Bicarbonate [mmol/L]*						
Day 1	35	26.73 (24.79, 27.31)	28.80 (27.70, 30.03)	0.84 (0.70–0.98)	5.27	< 0.001
Day 2	30	26.48 (26.13, 27.48)	29.88 (28.51, 30.88)	0.90 (0.79–1.01)	8.96	< 0.001
Day 3	26	26.40 (25.63, 26.88)	30.70 (29.48, 31.93)	0.94 (0.84–1.03)	15.67	< 0.001
Day 4	26	27.05 (25.60, 29.05)	31.10 (26.55, 31.83)	0.73 (0.51–0.94)	2.70	0.04
Day 5	26	27.15 (26.53, 27.80)	29.30 (27.03, 31.00)	0.68 (0.44–0.92)	2.13	0.13
Day 6	23	27.70 (26.49, 28.58)	28.90 (28.20, 31.05)	0.73 (0.51–0.96)	2.79	0.04
*pCO*^*2*^* [kPa]*						
Day 1	35	7.10 (6.54, 7.63)	7.32 (7.16, 8.36)	0.67 (0.47–0.87)	2.04	0.09
Day 2	30	6.28 (5.86, 7.05)	7.40 (7.12, 7.86)	0.78 (0.58–0.98)	3.57	0.01
Day3	26	5.90 (5.43, 6.50)	7.48 (6.84, 7.88)	0.80 (0.57–1.03)	4.03	0.01
Day4	26	5.64 (5.16, 7.15)	7.31 (6.71, 7.74)	0.73 (0.48–0.99)	2.79	0.07
Day5	26	6.17 (5.31, 7.45)	6.82 (6.42, 7.34)	0.63 (0.37–0.90)	1.70	0.31
Day6	23	6.12 (5.34, 8.38)	6.97 (6.56, 7.38)	0.55 (0.25–0.84)	1.22	0.74
*pH value*						
Day 1	35	7.31 (7.28, 7.34)	7.31 (7.29, 7.34)	0.51 (0.30–0.72)	1.05	0.91
Day 2	30	7.36 (7.30, 7.40)	7.35 (7.32, 7.38)	0.48 (0.24–0.71)	0.91	0.83
Day 3	26	7.38 (7.32, 7.42)	7.35 (7.31, 7.41)	0.46 (0.21–0.72)	0.85	0.76
Day 4	26	7.42 (7.33, 7.44)	7.34 (7.30, 7.40)	0.33 (0.09–0.57)	0.49	0.16
Day 5	26	7.41 (7.33, 7.45)	7.35 (7.31, 7.41)	0.34 (0.10–0.58)	0.52	0.18
Day 6	23	7.38 (7.28, 7.43)	7.35 (7.31, 7.39)	0.44 (0.15–0.73)	0.79	0.67
*Tidalvolume/kg PBW [ml]*						
Day 1	35	7.56 (6.32, 7.96)	6.75 (5.61, 7.57)	0.35 (0.16–0.55)	0.55	0.14
Day 2	30	7.91 (6.15, 9.28)	7.05 (5.88, 7.91)	0.33 (0.11–0.55)	0.49	0.12
Day 3	26	8.26 (6.30, 9.44)	7.23 (5.77, 8.38)	0.40 (0.14–0.66)	0.67	0.42
Day 4	26	8.99 (6.88, 9.61)	6.69 (5.79, 7.63)	0.28 (0.06–0.50)	0.39	0.05
Day 5	26	8.03 (6.36, 9.81)	7.78 (6.85, 8.82)	0.42 (0.15–0.70)	0.72	0.54
Day 6	23	7.21 (6.32, 8.85)	7.37 (6.43, 7.79)	0.45 (0.15–0.75)	0.82	0.74
*Tidal volume* < *8 ml/kg PBW [mL]*						
Day 1	35	11 (69%)	15 (79%)	–	–	0.34
Day 2	30	7 (50%)	12 (75%)	–	–	0.19
Day 3	26	5 (45%)	10 (67%)	–	–	0.35
Day 4	26	4 (36%)	12 (80%)	–	–	0.03
Day 5	26	5 (45%)	9 (60%)	–	–	0.61
Day 6	23	6 (60%)	10 (77%)	–	–	0.53
*Safety endpoints*						
Overall ICU mortality	35	13 (81%)	11 (58%)	–	–	0.13
Mortality during study	35	5 (31%)	4 (21%)	–	–	0.64
30 day mortality	35	10 (63%)	8 (42%)	–	–	0.26
Severe acidosis	35	3 (19%)	5 (26%)	–	–	0.64
Severe alkalosis	35	1 (6.3%)	0 (0%)	–	–	0.36
Hypernatremia	35	1 (6.3%)	3 (16%)	–	–	0.50
Severe hypernatremia	35	1 (6.3%)	0 (0%)	–	–	0.36
Severe hypocalcemia	35	1 (6.3%)	0 (0%)	–	–	0.36
Severe hypercalcemia	35	0 (0%)	0 (0%)	–	–	
Severe hypokalemia	35	0 (0%)	0 (0%)	–	–	
Severe hyperkalemia	35	0 (0%)	2 (11%)	–	–	0.40
Severe hypophosphatemia	35	4 (25%)	3 (16%)	–	–	0.59
Filter clotting	35	0 (0%)	1 (5.3%)	–	–	> 0.99
Citrate accumulation	35	1 (6.3%)	2 (11%)	–	–	> 0.99

^1^Number of patients included in the analysis. The distribution per group can be found in Supplemental Table 5. ^2^Values are median (IQR) or N = number (%), ^3^Relative effect *p* (Brunner-Munzel test), ^4^Confidence interval, ^5^Relative effect p/(1-p) ^6^Continuous variable using the Brunner-Munzel Test and categorical variable using the Boschloo's Exact Test, PBW: predicted body weight; pCO2: Carbon dioxide partial pressure; ICU = intensive care unit, Severe acidosis (pH < 7.15), Severe alkalosis (pH > 7.55), Hypernatremia (> 150 mmol/L), Severe hypernatremia (> 155 mmol/l), Severe hypocalcemia (iCa2 +  < 0.8 mmol/l), Severe hypercalcemia (iCa2 +  > 1.5 mmol/L), Severe hypokalemia (< 2.5 mmol/L), Severe hyperkalemia (> 6.5 mmol/L), Severe hypophosphatemia (< 0.5 mmol/L)

**Fig. 3:**
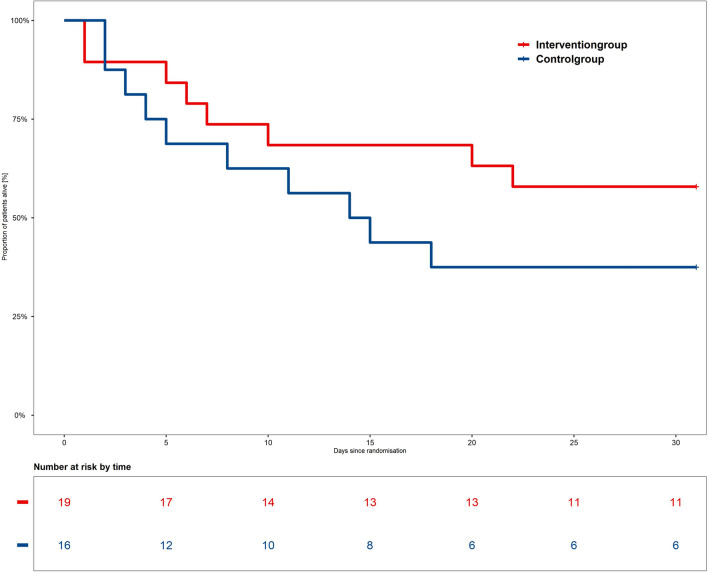
**Survival rate over 30 days.** From day 3 onwards, the survival rate remained consistently higher in the intervention group throughout both the six-day study period and the 30-day observation period. At the end of the study period of six days, 79% of individuals in the intervention group were alive as compared to 69% in the control group. After 30 days, the survival proportion remained higher in the intervention group, with 58% surviving, as opposed to 37% in the control group. However, the log-rank test indicated that there was no relevant difference in terms of 30-day mortality between the groups with a *p*-value of 0.23

Safety endpoints did not differ between both groups. As shown in Table 2 mortality was slightly higher in the control group as compared to the intervention group at all predefined time points: mortality during the study period of six days: 5/16 (31%) in the control group vs. 4/19 (21%) in the intervention group (*p* = 0.64); 30-day mortality 10/16 (63%) in the control group vs. 8/19 (42%) in the intervention group (*p* = 0.26); overall mortality in the intensive care unit 13/16 (81%) in the control group vs. 11/19 (58%) in the intervention group (*p* = 0.13). The survival rate over 30 days can be found in Fig. 3.

Although there were no relevant differences concerning severe acid–base or electrolyte disorders, sodium concentrations were higher in the intervention group as compared to the control group from day 1 to day 6, without severe hypernatremia (> 155 mmol/L) in the intervention group. This difference was greatest on day 2 with 146.15 mmol/L (IQR 145.48, 147.65) vs. 143.90 mmol/L (IQR 143.00, 144.40), *p* = 0.001, while by day 6, the difference was barely noticeable, with 144.90 mmol/L (IQR 143.90, 145.40) in the intervention group and 143.55 mmol/L (IQR 142.18, 144.70) in the control group (*p* = 0.03) (Supplemental Table 2). Severe hypernatremia occurred in only one case in the control group, but not in the intervention group (Table 2).

Events of filter clotting and citrate accumulation were similar between both groups. In one out of 16 patients (6.3%) in the control vs. in two out of 19 patients (11%) in the intervention group a citrate accumulation was observed (Table 2).

At the beginning of the study bilirubin levels were already higher in the intervention group as compared to the control group (1.14 mg/dL (IQR 0.53, 2.30) vs. 0.70 mg/dL (IQR 0.33, 1.21)) (Table 1). During the course of the study, the levels diverged starting from day two. On day 6, a similar ratio was observed as compared to the time of randomization, with 1.48 mg/dL (IQR 0.74, 2.84) vs. 0.73 mg/dL (IQR 0.49, 0.87), *p* = 0.03. (Supplemental Table 2). Individual patient data on bilirubin are included in Supplemental Table 3.

**Fig. 4:**
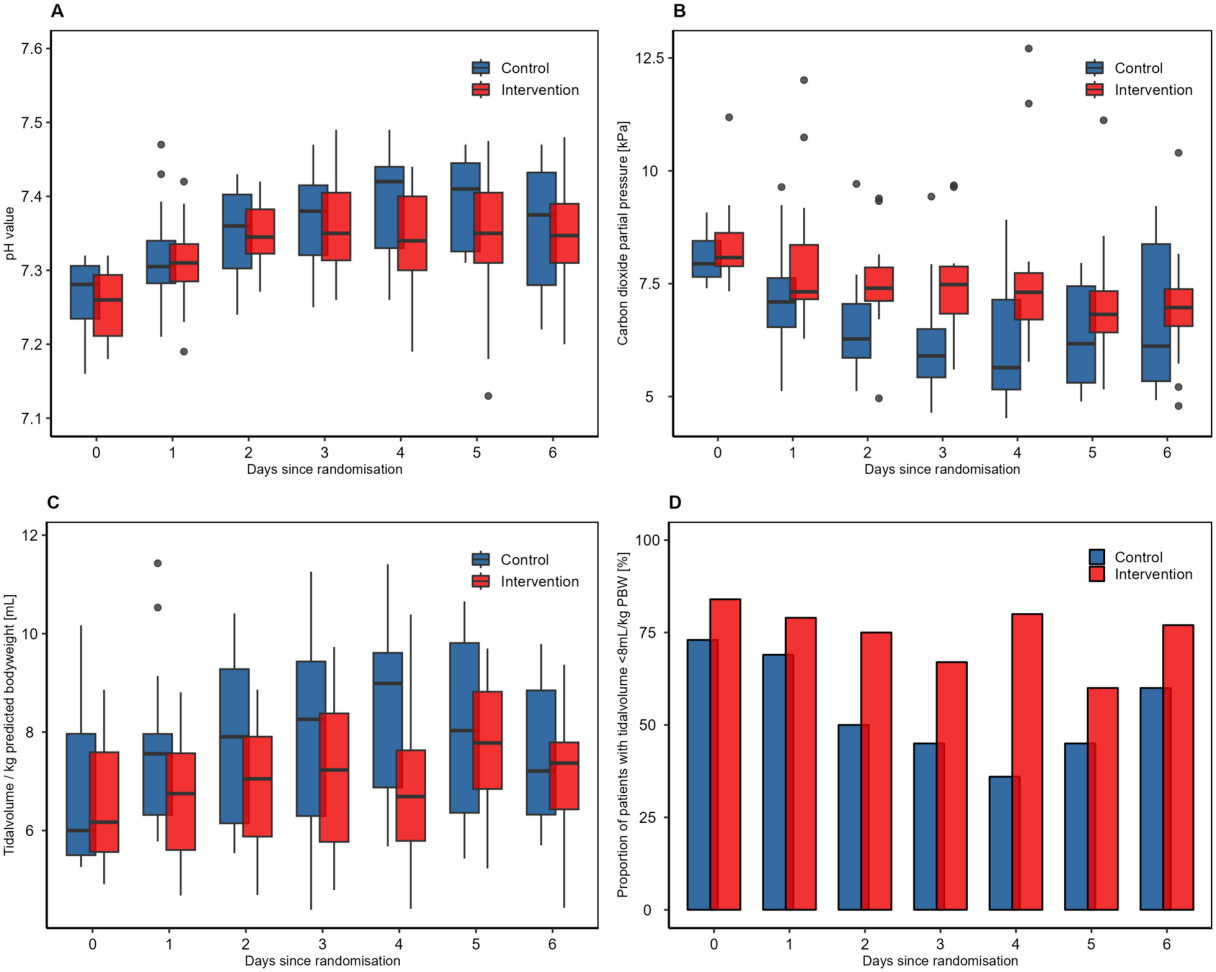
**Carbon dioxide level, pH and respiratory settings over the study period. A** pH value over the study period: Median pH value was similar between the two groups. Outliers are displayed as dots (IQR method). **B** Carbone dioxide partial pressure over the study period: Median pCO_2_ was higher in the intervention group. Outliers are displayed as dots (IQR method). **C** Tidalvolume/kg predicted bodyweight over the study period: Median tidalvolume/kg PBW was lower in the intervention group. Outliers are displayed as dots (IQR method). **D** Tidalvolume < 8 mL/kg predicted bodyweight during study period: Proportion of patients with tidalvolume < 8 mL/kg predicted bodyweight, in the intervention group more patients had a tidalvolume < 8 mL/kg predictes bodyweight as compared to the control group

Concerning clinical endpoints, the blood flow on RCA-CKRT was higher in the intervention group (intervention group 120 ml/min vs. control group 100 ml/min). The dialysate flow was similar in both groups (2000 ml/h) (Supplemental Table 2). The bicarbonate concentrations at 12-h intervals are provided in Supplemental Table 4. The pCO_2_ values were higher in the intervention group with considerable differences on days two and three (day 3: control group pCO_2_ = 5.90 kPa (IQR 5.43; 6.50), intervention group pCO_2_ = 7.48 kPa (IQR 6.84; 7.88), *p* = 0.01. Despite the higher pCO_2_ levels, there was no noteworthy difference concerning the pH-value (Table 2; Fig. 4A and B). The tidal volumes were lower in the intervention group as compared to the control group (Table 2). Furthermore, from day two on, the respiratory minute volume/kg PBW was numerical lower in the intervention group as compared to the control group (Supplemental Table 2). Lung protective ventilation defined as tidal volume < 8 ml/kg PBW was applied more frequently throughout the entire study period in the intervention group. On day 4, the difference in the proportion of patients receiving lung protective ventilation was most pronounced with 12/15 (80%) in the intervention group vs. 4/11 (36%) in the control group, *p* =  < 0.03 (Table 2; Fig. 4D). Both, the IPP and the positive end-expiratory pressure (PEEP) were already higher in the intervention group at the time of study enrollment (Table 1). Throughout the study, this difference persisted, with distinctions observed on day one: median PEEP was 11.5 mBar (IQR 10.0, 14.0) in the control group versus 16.0 mBar (IQR 14.0; 17.0) in the intervention group (*p* =  < 0.01), and median IPP was 27.0 mBar (IQR 25.3; 27.0) in the control group versus 30.0 mBar (IQR 27.5; 32.0) in the intervention group, *p* =  < 0.01). However, the driving pressure did not differ between the groups. (Supplemental Table 2). Median Horrowitz-Index and norepinephrine dose were similar between the two groups (Supplemental Table 2). 8/19 (42%) patients in the intervention group as compared to 3/16 (19%) patients in the control group were discharged from the ICU after a median of 82 days (IQR 53; 116) in the intervention group respectively 35 days (IQR 33; 59) in the control group (Supplemental Table 2). The duration of mechanical ventilation in ICU-surviving patients was 76 days (IQR 49; 110) in the intervention group as compared to 34 days (IQR 30; 56) in the control group (Supplemental Table 2). Supplemental Fig. 2 illustrates the relative effect of the intervention on the bicarbonate concentration, pCO2, pH, and tidal volume per kilogram PBW.

## Discussion

In this randomized controlled pilot trial in hypercapnic ARDS patients undergoing RCA-CKRT, targeting a pCO_2_-tailored plasma bicarbonate concentration yielded higher systemic bicarbonate levels, mimicking effective metabolic compensation of respiratory acidosis. Tidal volumes were lower in the intervention group and lung protective ventilation was applied more often. There was no relevant difference concerning adverse events. Mortality was numerically higher in the control group as compared to the intervention group.

Current CKRT protocols do not account for the physiological renal compensation of respiratory acidosis and we are not aware of any clinical trials investigating the buffering of respiratory acidosis in ARDS patients, particularly not in patients with concurrent indication for CKRT. Existing reviews suggest a potential advantage of CKRT-based buffering over intravenous drug buffering due to its slow and continuous regulation of acid–base balance while controlling fluid balance [3, 16, 17].

Our findings show that a pCO_2_-adapted CKRT is feasible and safe. Furthermore, although not the primary endpoint, our results suggest that the intervention might help to facilitate lung protective ventilation in hypercapnic ARDS. Noteworthy, although pCO_2_ values were higher in the intervention group, pH values remained similar between the groups. This may in part be explained by the lower tidal volumes and lower respiratory minute volume in the intervention group, marking lung protective ventilation. Maintaining pH in a certain level is a common motivation to increase intensity of ventilation, and low bicarbonate has been identified as one factor leading to violation of lung-protective ventilation strategies [18].

Even though hypercapnia was rather moderate in our study population (median pCO_2_ at study inclusion around 8 kPa in both groups) it resembles pCO_2_ values in other ARDS trials with low tidal ventilation with pCO_2_ values of around 5.33–6.93 kPa [19, 20].

We cannot exclude, that the elevated pCO_2_ partly derived from increased bicarbonate levels implying an additional CO_2_ load. Prior studies addressing the question of a dialysis-derived additional CO_2_ load showed different results: Symreng et al. reported an excess CO_2_ burden during high-efficiency intermittent hemodialysis [21]. However, this might not apply to CKRT as the rate of buffer administration is much slower. In a randomized experimental trial involving hypercapnic pigs on a continuous hemodialysis, a higher bicarbonate concentration in the dialysate resulted in improved blood pH control without elevation in pCO_2_ levels [22].

By administration of a higher amount of citrate and therefore HCO_3-_ there is a possible risk of electrolyte and acid–base disorders, including metabolic alkalosis, hypernatremia (citrate is administered as trisodium citrate) or hypocalcemia (as citrate binds calcium) [23]. Although sodium concentrations were mildly higher in the intervention group, we did not observe any severe hypernatremia in the intervention group. There was no severe alkalosis or hypocalcemia in the intervention group either.

Furthermore, the higher amount of citrate might lead to an increased risk of citrate accumulation characterized by metabolic acidosis and reduced ionized calcium. Citrate accumulation was observed in one out of 16 patients (6.3%) in the control vs. in two out of 19 patients (11%) in the intervention group. This is slightly higher as compared to another study which reported citrate accumulation in 5% of patients—specifically 162 unselected ICU patients requiring CKRT [8]. In cases where citrate accumulation occurred, it was during episodes of severe sepsis and respiratory failure, which underscored the complexity and severity of the clinical scenarios faced. Not only is liver failure a known risk factor for citrate accumulation, but lactate kinetics also pose a significant risk, which can be challenging to predict [24]. Furthermore, the ability to metabolize citrate in the Krebs cycle is oxygen-dependent. Thus, the numerically higher incidence of citrate accumulation could in theory be partially explained by the relative hypoxemia in ARDS patients. We are not aware of any studies investigating the incidence of citrate accumulation in ARDS patients. As pointed out previously by Israni et al. it is important to remember that citrate, per se, is not toxic; rather, the accumulation signals the manifestation of an underlying severe medical condition [25]. In total we do not see an increased risk of citrate accumulation in our intervention.

Bilirubin levels were higher in the intervention group as compared to the control group at the beginning of the study (control group 0.70 mg/dL (IQR 0.33; 1.21) as compared to 1.14 mg/dL (IQR 0.53; 2.30) in the intervention group) and this difference became larger during the study. When examining the values of individual study patients, it becomes evident that the difference can be attributed to three patients who already had markedly elevated bilirubin levels at baseline which increased during the study. Regional citrate anticoagulation is not contraindicated even in the presence of liver failure, and a negative effect on liver function due to citrate has not been reported [26, 27]. Rather, careful monitoring for citrate accumulation is recommended.

Mortality rates were high in both the control and the intervention group. However, given the fact that all patients suffered from multiorgan failure with ARDS and AKI requiring KRT mortality seems comparable to other studies reporting mortality rates between 55 and 73% [28, 29]. The survival rate in the intervention group was noticeably higher after 30 days, with 58% as compared to 37%. However, it is important to emphasize that the study was not powered for mortality, and the observed effect could be incidental. Such a survival benefit is unexpected, even when considering the potential advantages attributed to a more lung-protective ventilation strategy. Nevertheless, the lower mortality in the intervention group supports the impression that the intervention of a pCO_2_-adapted CKRT is safe.

Duration of mechanical ventilation and duration of stay in the ICU was longer in the intervention group as compared to the control group. However, this might be attributable to the higher ICU-survival rate in the intervention group.

The amount of HCO_3-_ application via the RCA-CKRT, can be adjusted either via the dialysate flow or via the administered citrate which is metabolized in HCO_3-_ [7, 23]. In order to apply the same dialysate dose to the patients we decided to first adjust the citrate dose by change of the blood flow before reducing the dialysate flow. The blood flow was higher in the intervention group showing the successful increased application of citrate and therefore HCO_3-._ Worth noting is the relatively moderate intervention intensity in our study. We were able to achieve the targeted bicarbonate with only small adjustments in blood flow and therefore citrate application. The dialysate dose remained predominantly unchanged, demonstrating that our interventions did not compromise the overall quality of dialysis. This underscores the efficiency and practicality of our approach, showcasing its potential to possibly influence clinical outcomes without significant alterations in the dialysis procedure.

Extracorporeal devices can remove CO_2_ in critically ill patients such as ECMO or less invasive extracorporeal carbon dioxide removal (ECCO_2_R)-devices that can partly be integrated in the CKRT [1, 30]. These devices are efficient in reducing CO_2,_ optimizing pH and enabling (ultra) lung protective ventilation [31]. In these patients buffering of respiratory acidosis is not necessary. However, not all patients qualify for ECMO-therapy, until now clinical studies failed to show a clinical benefit of ECCO_2_R-devices and extracorporeal devices are not free of side effects especially bleeding but also hemolysis, thrombotic events or access site comlications [1, 30]. In contrast to ECMO, current guidelines recommend against the use of ECCO_2_R-devices in ARDS patients outside of RCTs [1]. Furthermore, filters for extracorporeal CO_2_ removal are expensive and not generally available. Metabolic buffering via the RCA-CKRT might be a simple, cost-effective and promising alternative.

Taken together this pilot study demonstrated several important findings regarding feasibility, applicability and safety: (i) pCO_2_ adapted CKRT is feasible, leading to a significant increase in bicarbonate and better control of pH in ARDS patients; (ii) the intervention is safe in terms of electrolyte control and mortality. Additionally, our pilot trial provides valuable information for a potential follow-up trial. First, the cohort of ARDS patients showed a potential risk for citrate accumulation. We do not consider this to be a risk imposed by our intervention, but rather believe that this is an expression of the severity of illness of the included cohort. Nevertheless, in a follow-up trial we have to acknowledge the probable higher incidence of citrate accumulation in hypoxemic ARDS patients and would define a therapy resistant lactic acidosis as an exclusion criteria, as this represents a risk for reduced citrate metabolism and could lead to citrate accumulation. Second, we believe that a follow up trial should be designed using lung protective ventilation as primary endpoint. Therefore, a protocol for the adjustment of mechanical ventilation in the intervention group should be incorporated in a follow-up study.

This trial has several strengths and limitations. To our knowledge this is the first study investigating the effects of a pCO_2_-adapted CKRT providing important data on an easy and promising clinical application especially in patients without indication for extracorporeal lung support. As a pilot trial the study was designed to primarily investigate feasibility and safety and was therefore not powered on clinical endpoints. Whether the positive effects on lung protective ventilation translate into improved clinical endpoints therefore needs to be evaluated in larger prospective studies. As blinding was not feasible, a possible performance and detection bias cannot be excluded. As an open label trial a cointervention bias especially concerning lung protective ventilation strategies cannot be excluded. However, our trial did not include any protocol that mandated respirator setting and, as a local peculiarity in our university clinic, the dialysis team is exclusively responsible for the CKRT and its adjustments and not the treating physician himself. This allowed us to provide an environment where the cointervention bias could be excluded as far as it was possible in an open-label study. Due to the predominantly male composition of study participants and the pilot nature with limited patient numbers, the relevance of the findings for female patients may be restricted, and subgroup analyses were not conducted. This study was performed in hypercapnic ARDS patients. It would be interesting to expand the intervention to a broader patient group with hypercapnia, including patients with chronic obstructive pulmonary disease (COPD).

## Conclusion

In this pilot-trial the use of a pCO_2_ adapted continuous hemodialysis in hypercapnic ARDS patients was feasible and appeared safe, warranting its evaluation to enable lung protective ventilation and improve clinical outcomes.

### Supplementary Information

Additional file1 (PDF 1180 KB)

## Abbreviations

PBW: Predicted body weight
ARDS: Acute respiratory distress syndrome
pCO_2_: Carbon dioxide partial pressure
HCO_3-_: Bicarbonate
AKI: Acute kidney injury
CKD: Chronic kidney disease
CKRT: Continuous kidney replacement therapy
RCA: Regional citrate anticoagulation
TRIS: Trometamol
ICU: Intensive care unit
ECMO: Extracorporeal membrane oxygenation
IPP: Inspiratory plateau pressure
FMC: Fresenius medical care
SOFA: Sequential organ failure assessment
DRKS: German registry of clinical trials
IQR: Interquartile range
PEEP: Positive end-expiratory pressure
ECCO2R: Extracorporeal carbon dioxide removal
