# Supplementary material for: Standard vs. carbone dioxide adapted kidney replacement therapy in hypercapnic ARDS patients: a randomized controlled pilot trial (BigBIC)
Source: Crit Care. 2024 Jun 11;28:198. doi: 10.1186/s13054-024-04979-z (PMC11167756; doi:10.1186/s13054-024-04979-z)
Supplement: Supplementary file 1 — Additional file1 (PDF 1180 KB) [file 13054_2024_4979_MOESM1_ESM.pdf]

Standard vs. carbone dioxide adapted kidney replacement therapy in hypercapnic ARDS patients: A randomized controlled pilot trial (BigBIC)

Supplementary material

Table of Contents:

Study Protocol	2-11
Supplemental Figure 1: Scheme of pCO2 adapted CKRT	12
Supplemental Table 1: Amount of citrate added relative to blood-flow	13
Supplemental Table 2: Additional outcomes of the study participants	14-15
Supplemental Table 3: Individual bilirubin levels in mg/dL	16
Supplemental Table 4: 12-hour bicarbonate concentrations	17
Supplemental Figure 2: Relative effects of the intervention on HCO3, pCO2, pH and tidalvolume	18
Supplemental Table 5: Distribution into groups	19

Study protocol

The BIG-BIC-Study

Carbon dioxide partial pressure (pCO_2) adapted continuous hemodialysis in lung-protective ventilated ARDS patients with renal failure requiring dialysis.

SPONSOR: Charité - Universitätsmedizin, Berlin

Representative of the Sponsor:

PD Dr. Philipp Enghard
Charité – Universitätsmedizin Berlin
Department of Nephrology and Medical Intensive Care
Augustenburger Platz 1, 13353 Berlin
Phone: +49 (0) 30 450 614 016
Email: philpp.enghard@charite.de

Table of Contents

1. Protocol Synopsis 4
    1.1 Title of study 4
    1.2 Study design 4
    1.3 Study centers 4
    1.4 Intervention 4
        1.4.1 Reference exposure 4
        1.4.2 Investigational exposure 4
    1.5 Study period 4
    1.6 Study purpose 4
    1.7 Methods 4
    1.8 Objectives and Outcome measures 4
        1.8.1 Primary endpoint: Efficacy 4
        1.8.2: Secondary endpoints 4-5
    1.9 Population 5
    1.10 Inclusion/Exclusion criteria 5
        1.10.1 Inclusion criteria 5
        1.10.2 Exclusion criteria 5
    1.11 Sample Size 5
2. Introduction 6
3. Study design 6
    3.1 Intervention scheme 6-7
    3.2 Study endpoints 7
        3.2.1 Primary endpoint: efficacy 7
        3.2.2 Sedondary endpoints 7
    3.3. Randomization 7
    3.4 Blinding assessment 8
4. Study assessment 8-9
5. Safety assurance 9
    5.1 Safety of the procedure 9
    5.2 Risk mitigation 9
    5.3 Safety assessment 9-10
    5.4 Criteria for study discontinuation 10
6. Data management 10
7. Statistical analysis 10
8. Ethics and good clinical practice 10
    8.1 Good clinical practice 10
    8.2 Ethics committee 10
    8.3 Informed consent 10-11
    8.4 Declaration of Helsinki 11
9. Investigator signature 11
10. References 11

1 Protocol Synopsis

1.1 Title of study

The BIG-BIC-Study - Carbon dioxide partial pressure (pCO_2) adapted continuous hemodialysis in lung-protective ventilated ARDS patients with renal failure requiring dialysis.

1.2 Study design:

Single center, prospective, randomized pilotstudy investigating the effect of carbon dioxide partial pressure (pCO_2) adapted continuous hemodialysis in lung-protective ventilated ARDS patients with renal failure requiring dialysis.

1.3 Study centers

Mixed intensive care units of the Charité University medicine Berlin

1.4 Intervention

Carbon dioxide partial pressure (pCO2) adapted continuous hemodialysis with citrate anticoagulation in lung-protective ventilated ARDS patients with renal failure requiring dialysis.

1.4.1 Reference exposure

Standard treatment with target HCO_3 to 24mmol/l

1.4.2 Investigational exposure

Standard of care and target HCO_3 to ((pCO_2-45mmHg)/10mmHg)*4mmol/l + 24mmol/l

1.5 Study period

Intervention for 6 days. Filter exchange every 72 hours.
Patient surveillance for the primary endpoint (efficacy) and secondary endpoints for 6 days. Follow up for mortality for 30 days or until ICU discharge.

1.6 Study purpose

To evaluate the effect of carbon dioxide partial pressure (pCO2) adapted continuous hemodialysis with citrate anticoagulation to support lung-protective ventilation in ARDS patients with renal failure requiring dialysis

1.6.1 Primary hypothesis

By adjusting the blood-to-dialysate ratio, HCO_3 can be safely raised in accordance with pCO_2 in lung-protective ventilated ARDS patients treated with citrate anticoagulated continuous hemodialysis (target HCO_3 = ((pCO2-45mmHg)/10mmHg)*4mmol/l + 24mmol/l)))

1.6.2. Secondary hypotheses

The pCO_2 adapted bicarbonate target may facilitate or enable lung protective ventilation, as acidosis is often the limiting factor. Of note are the tidal volumes in both groups measured every 12 hours and 72 hours after study inclusion.

1.7 Methods

This is a randomized, prospective single center trial investigating the effect of carbon dioxide partial pressure (pCO2) adapted continuous hemodialysis with citrate anticoagulation to support lung-protective ventilation in ARDS patients with renal failure requiring dialysis. Patients with acute kidney injury requiring dialysis (AKI-KRT) will be randomized to receive either standard of care with targeted bicarbonate to 24 mmHg or standard of care and targeted bicarbonate to (CO_2-45mmHg)/10*4 mmHg + 24). The intervention will be carried out for 6 days with a filter exchange every 72 hours. Patients with CRRT <48h after study inclusion will be excluded from the final analysis.

1.8 Objectives and Outcome measures

1.8.1 Primary endpoint: Efficacy

Bicarbonate concentration 72hours after study inclusion in both groups.

1.8.2 Secondary endpoints:

Efficacy: Kinetics of HCO-concentrations measures every 12 hours in both groups.

Safety: differences of adverse events in both groups during the time of intervention of 6 days:

- Mortality
- Severe acidosis (pH< 7,15)
- Severe alkalosis (pH >7,55)
- hypernatremia (>155mmol/l)
- severe hypo/hypercalcemia (iCa^{2+} <0,8 mmol/l or >1,5 mmol/l)
- severe hypo/hyperkalemia (<2,5mmol/l >6,5mmol/l)
- severe hypophosphatemia (<0,3mmol/l)
- Filterclotting
- Citrataccumulation

Median of the following parameters over the intervention period of 6 days:
-pH
-tidal volumes
-driving pressure
-peak pressure
-respiratory minute volume

and
-catecholamine dose (max daily day 1-6)
-duration of mechanical ventilation (days)
-mortality (30 days or until ICU-discharge
-SOFA-score (day 1-6)
-blood-to-dialysis-ratio

1.9 Population

The study population will consist of patients with acute respiratory distress syndrome (ARDS) of all cause without time restriction and indication for continuous hemodialysis who are consecutively admitted to one of the ICUs of the study center and fulfilling the following inclusion criteria.

1.10 Inclusion/Exclusion criteria

1.10.1 Inclusion criteria:

- Any gender aged >18 years
- ARDS (any cause) defined by Berlin Definition (1) (modified, no time restriction).
- CRRT with citrate anticoagulation
- Invasive mechanical ventilation
- Hypercapnia defined as pCO_2 > 55mmHg with tidals >4 ml/kg and respiratory rate ≥12/min

1.10.2 Exclusion criteria:

- ECMO
- TRIS-buffering
- Lactic acidosis > 80mg/dl; Liver failure (Bili > 8 and Quick < 50);
-

1.11 Sample size

The study is a pilot study.

The Wilcoxon-Mann-Whitney rank sum test for continuous outcomes and the nQuery version 8.7.2 program were used for case design.
In a randomized controlled trial, the median bicarbonate in 48 patients with normal liver function at dialysis initiation was 20.9 mmol/l +/- 4.8. 72 h after dialysis initiation, the median bicarbonate was 25.2 mmol/l +/- 2.4. (2)

A median of 24 mmol/l is assumed in the control group (at time 72 hours after dialysis initiation), and a median of 32 mmol/L is assumed in the intervention group (corresponding to a $paCO_2$ of 65 mmHg according to the formula). Since we assume a larger standard deviation in the intervention group than in the above study, because the patients have different target bicarbonate levels, a common standard deviation of 8 was assumed. To demonstrate this effect with a power of 80% at a significance level of 5%, a case number of 20 per group is needed.

Statistical advice is provided by:
Mareen Pigorsch, Biostatistician
Institute for Biometry and Clinical Epidemiology

2 Introduction

Lung protective ventilation with low tidal volumes (4-6 ml/kgKG) and low inspiratory pressures (peak pressure <30 cmH2O) are part of the standard therapy in patients with Acute Respiratory Distress Syndrome (ARDS) according to national and international guidelines(3-5). In low tidal ventilation moderate CO2 increase is tolerated in favor of lung protection (permissive hypercapnia). In individuals with normal renal function CO_2 retention is compensated via increased bicarbonate reabsorption in the kidney to avoid higher-grade pH shifts. Adequate compensation in chronic respiratory acidosis is equivalent to a factor of 0.4 (HCO2 increase per CO2 increase) (6). Acute renal failure is a common complication in ARDS patients and affects approximately 38-68% of patients(7, 8). Both in these patients and in patients with chronic kidney disease the ability to balance the acid-base budget is impaired. To avoid a pronounced pH shifts (pH<7.2) and associated negative effects, buffer substances (e.g., sodium bicarbonate, tromethamol (TRIS)) are used. Regarding drug buffering in respiratory acidosis, data is uncertain. Some studies, particularly on intravenous bicarbonate administration, have shown adverse effects such as volume overload, hyperosmolarity, and hypocalcemia. In addition, there is concern of possible additive CO_2 load.
Adjusting the blood-to-dialysate ratio can influence systemic bicarbonate in patients on citrate anticoagulated continuous hemodialysis.
Typically, values between 22 and 24 mmol/l are targeted regardless of pCO_2. Randomized controlled studies on target bicarbonate levels in patients with continuous dialysis in ARDS are lacking.
A pCO_2-adapted bicarbonate target in continuous hemodialysis could avoid adverse effects via slow and continuous regulation of acid-base balance while controlling fluid balance.(9) By changing the blood-to-dialysate ratio, according to the expected physiological renal output, in ARDS patients with renal failure requiring dialysis, lung-protective ventilation could be facilitated or enabled, as acidosis is often the limiting factor.

3 Study design

Randomized, prospective single center pilot trial investigating the effect of carbon dioxide partial pressure (pCO2) adapted continuous hemodialysis with citrate anticoagulation to support lung-protective ventilation in ARDS patients with renal failure requiring dialysis.

3.1 Intervention scheme

All patients >18 years with ARDS of all cause without time restriction and indication for continuous hemodialysis who are consecutively admitted to one of the ICUs of the study center and fulfilling the inclusion criteria are eligible for the study. After written informed consent 1:1 randomization is done using sealed envelopes. Patients will be randomized to receive either standard of care with targeted bicarbonate to 24 mmHg or standard of care and targeted bicarbonate to ((pCO2-45mmHg)/10mmHg)*4mmol/l + 24mmol/l)). The intervention will be carried out for 6 days with a filter exchange every 72 hours. Therapy is done open-label as blinding is not possible. Data will be collected from the electronical health record. No additional data other than routine clinical data will be collected.
Primary endpoint is the bicarbonate concentration 72hours after study inclusion in both groups.

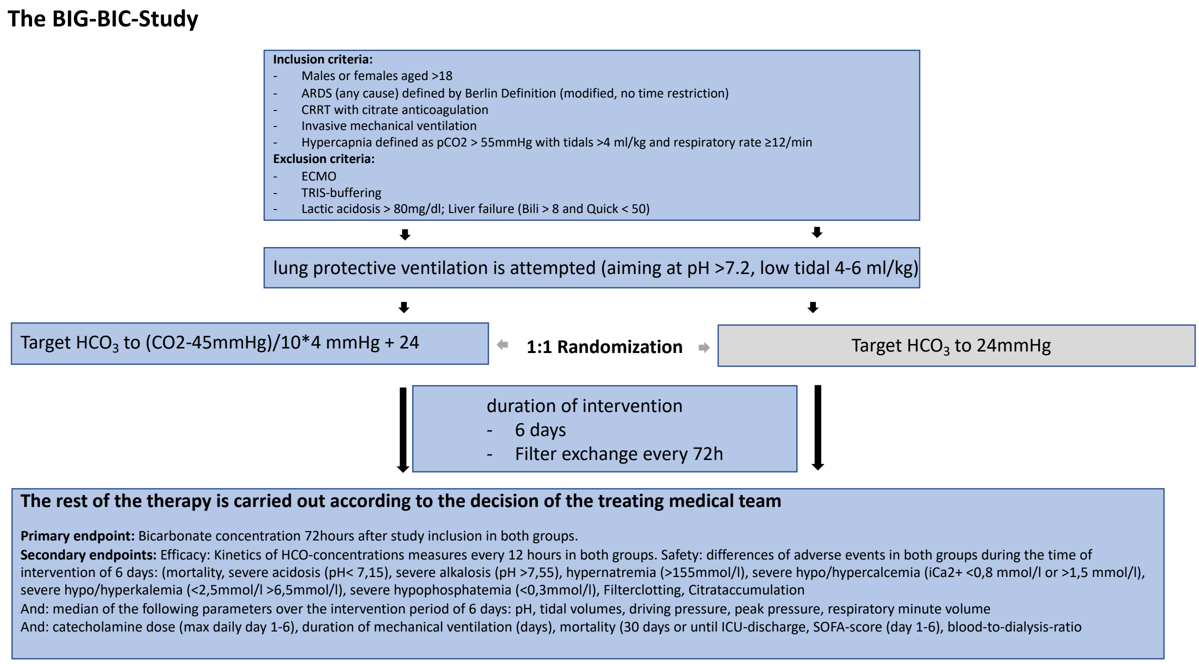

Figure 1: Trial Flow Chart

3.2 Study Endpoints

3.2.1 Primary endpoint: Efficacy
Bicarbonate concentration 72hours after study inclusion in both groups.

3.2.2 Secondary endpoints:
Efficacy: Kinetics of HCO-concentrations measures every 12 hours in both groups.

Safety: differences of adverse events in both groups during the time of intervention of 6 days:

- Mortality
- Severe acidosis (pH< 7,15)
- Severe alkalosis (pH >7,55)
- hypernatremia (>155mmol/l)
- severe hypo/hypercalcemia (iCa2+ <0,8 mmol/l or >1,5 mmol/l)
- severe hypo/hyperkalemia (<2,5mmol/l >6,5mmol/l)
- severe hypophosphatemia (<0,3mmol/l)
- Filterclotting
- Citrataccumulation

Median of the following parameters over the intervention period of 6 days:
-pH
-tidal volumes
-Driving pressure
-peak pressure
-respiratory minute volume

and
-catecholamine dose (max daily day 1-6)
-duration of mechanical ventilation (days)
-mortality (30 days or until ICU-discharge
-SOFA-score (day 1-6)
-blood-to-dialysis-ratio

3.3 Randomization

The randomization will be performed 1:1 using sealed envelopes.

3.4 Blinding assessment

Blinding is not feasible as a clinical intervention is done. The statistician will be blinded.

4 Study assessment

All laboratory parameters will be analyzed locally.

Baseline variables:

- Age
- Sex
- Comorbidities
  - Hypertension
  - Diabetes
  - Coronary heart disease
  - COPD
  - History of malignancy
  - Chronic kidney disease
  - Immunosuppression
  - Other

At inclusion

- pH
- HCO3
- paCO2
- Pa02
- Fi02
- PEEP
- Plateau pressure
- Delta P
- Tidal voluminal
- Respiratory minute volume
- Dose of Noradrenaline
- Other Vasopressors and their doses
- Glucocorticoids
- SOFA
- APACHE (ICU admission)

Outcome parameters

- HCO3 (daily median d 1-6)
- pC02(daily median d 1-6)
- pH (daily median d 1-6)
- Tidal volumes (daily median d 1-6)
- Delta-p (daily median d 1-6)
- plateau pressure (daily median d 1-6)
- respiratory minute volume (daily median d 1-6)
- time on ventilator (days)
- mortality (ICU-discharge or 30-day)
- Dose of Noradrenaline (max d 1-6)
- Other Vasopressors and their doses (max d 1-6)
- daily SOFA (d 1-6)
- Blood to dialysis ratio (daily d 1-6)

Safety endpoints

- Mortality
- Severe acidosis (pH< 7,15)
- Severe alkalosis (pH >7,55)
- hypernatremia (>155mmol/l)
- severe hypo/hypercalcemia (iCa2+ <0,8 mmol/l or >1,5 mmol/l)
- severe hypo/hyperkalemia (<2,5mmol/l >6,5mmol/l)
- severe hypophosphatemia (<0,3mmol/l)
- Filterclotting
- Citrataccumulation

Serious adverse events

- Cardiac arrest
- Severe bleeding
- Severe allergic reaction
- Death
- Other

Laboratory Data (day 1-6)

- CRP
- PCT
- Troponin
- D-Dimer
- LDH
- Creatinine
- ALT
- AST
- gGT
- Bilirubine
- CK
- total serum calcium
- Albumin
- blood urea nitrogen (BUN)
- INR

5 Safety assurance

5.1 Safety of the procedure

The purpose of this study is to demonstrate the safety and efficacy of pCO2-adapted continuous dialysis therapy (CRRT) with citrate anticoagulation in ARDS patients with hypercapnia to facilitate lung protective ventilation. No additional substances or drugs are administered; only the ratio of blood flow to dialysate flow is subject of the intervention.
Uncertainty exists as to whether raising bicarbonate is safe in respiratory acidosis. The data on this is inconsistent. Some studies, particularly on intravenous bicarbonate administration, have shown adverse effects such as volume overload, hyperosmolarity, and hypocalcemia. In addition, there is concern of possible additive CO_2 loading. Raising the bicarbonate concentration via adjustment of the blood-to-dialysate ratio on continuous hemodialysis may have advantages in this regard, as it is much slower and any adverse effects such as volume overload can be addressed directly.(9) Moreover, the adapted bicarbonate target in the intervention group corresponds to the expected physiological adaptation and thus appears rather moderate.
The participating patients receive continuous intensive medical care and monitoring, so that dangerous shifts in acid/base and electrolyte levels can be quickly detected. Moreover, since patients are on CRRT anyway, these disturbances would be quickly reversible and well treatable.
If the intervention proves safe, it could be used to treat respiratory acidosis, as this is often the limiting factor in lung protective ventilation.
This could enable to use lower ventilatory pressures to help provide lung protective ventilation in ARDS.

5.2 Risk mitigation

After inclusion in the study, the patients are monitored by the attending physicians of the participating intensive care units until their discharge and are visited daily by the study team until the end of the intervention. Continuous intensive care monitoring with serial arterial blood gas analyses and daily routine laboratory tests will be performed as part of the intensive care therapy.
By excluding patients with lactic acidosis (> 80mg/dl) and/or liver failure (Bili > 8 and Quick < 50) the potential additive risk of citrate accumulation will be minimized by changing the blood to dialysate ratio.

5.3 Safety assessment

The treating physicians identify adverse events and document them in the patient record during routine clinical practice. The study physicians or study nurses document these promptly in the paper-based CRF. Data on mortality and adverse events are continuously submitted to the study management and reviewed by them for potential safety concerns. If a relevant difference in mortality or a clustered occurrence of adverse events in one of the two groups becomes apparent, independent medical and biostatistically trained colleagues will be involved to advise on the continuation of the study.

5.4 Criteria for Study discontinuation

Stopping rules for the individual patient is patient wish, suspected or proven citrate accumulation with switch to citrate-free continuous hemodialysis, initiation of ECMO, initiation of TRIS buffering.
Stopping rules for the whole trial are safety concerns due to the ongoing surveillance of mortality and serious adverse events by the investigators in consult with independent medical and biostatistically trained colleagues.

6 Data management

Routine clinical data are continuously documented in the patient file. Data collection is carried out by means of automated recording (COPRA): There is a local digital connection to the KISS system. Then the plausibility check of the data, the printout as paper CRF, the validation by the study physician and the dispatch to the study management takes place.
No samples or data are obtained outside of routine clinical practice.
Patient data will only be collected and stored pseudonymously. The study physician generates a pseudonym for each new patient to be created according to the guidelines listed below. Patients are uniquely assigned via the patient identification list, which must be stored securely. Only the physicians or study nurses involved in the study have access to the patient identification list.
The results of the above-mentioned study, compiled according to groups, will be published in a medical journal without the possibility of reference to the respective person and regardless of whether the results are positive or negative. The data recorded outside the medical record will be destroyed by the end of the study.

7 Statistical analysis

The study is a pilot study.

The Wilcoxon-Mann-Whitney rank sum test for continuous outcomes and the nQuery version 8.7.2 program were used for case design.
In a randomized controlled trial, the median bicarbonate in 48 patients with normal liver function at dialysis initiation was 20.9 mmol/l +/- 4.8. 72 h after dialysis initiation, the median bicarbonate was 25.2 mmol/l +/- 2.4.(2)

A median of 24 mmol/l is assumed in the control group (at time 72 hours after dialysis initiation), and a median of 32 mmol/L is assumed in the intervention group (corresponding to a $paCO_2$ of 65 mmHg according to the formula). Since we assume a larger standard deviation in the intervention group than in the above study, because the patients have different target bicarbonate levels, a common standard deviation of 8 was assumed. To demonstrate this effect with a power of 80% at a significance level of 5%, a case number of 20 per group is needed.

Primary and Secondary endpoints will be evaluated with adequate models (T-Test/Mann-Witney-U-Test/proportional hazards model /logistic regression) depending on distribution of endpoint variables.
All the analyses will be explorative and will not be controlled for multiple testing. Since missing data is not expected, imputation is not planned.
Blinding of the investigator and patient is not possible as a clinical intervention is done, statistician will receive a blinded data set, so that all analyses will be conducted blinded.

8 Ethics and Good Clinical Practice

8.1 Good Clinical Practice

This study must be carried out in compliance with the protocol and the principles of GCP. By signing this protocol, the investigator agrees to adhere to the instructions and procedures described in it and thereby to adhere to the principles of GCP to which it conforms.

8.2 Ethics Committee

This study was approved by the local ethics committee (EA2/101/21).

8.3 Informed consent

The investigator must explain to each patient the nature of the study, its purpose, the procedures involved, the expected duration, the potential risks and benefits involved and any discomfort it may entail. Each patient must be informed that participation in the study is voluntary and that he/she may withdraw from the study at any time and that withdrawal of consent will not affect his/her subsequent medical treatment or relationship with the treating physician. This informed consent should be given by means of a standard written statement, written in nontechnical language. The patient should read and consider the statement before signing and dating it and

should be given a copy of the signed document. If a patient is not able to give written informed consent himself, and a legal representative exists, the consent of the representative will be obtained.

8.4 Declaration of Helsinki

The investigator must conduct the trial in accordance with the principles of the Declaration of Helsinki.

9 Investigator Signature

I confirm that I have read this protocol and agree to conduct this study in accordance with all stipulations of the protocol and in accordance with ICH guidelines, GCP, and applicable local requirements.

Investigator

Date: ________

Signature: _________________________

Name (block letters): ______________________________

10 References

1. Ranieri VM, Rubenfeld GD, Thompson BT, Ferguson ND, Caldwell E, Fan E, et al. Acute respiratory distress syndrome: the Berlin Definition. JAMA. 2012;307(23):2526-33.
2. Slowinski T, Morgera S, Joannidis M, Henneberg T, Stocker R, Helset E, et al. Safety and efficacy of regional citrate anticoagulation in continuous venovenous hemodialysis in the presence of liver failure: the Liver Citrate Anticoagulation Threshold (L-CAT) observational study. Crit Care. 2015;19:349.
3. Brower RG, Matthay MA, Morris A, Schoenfeld D, Thompson BT, Wheeler A. Ventilation with lower tidal volumes as compared with traditional tidal volumes for acute lung injury and the acute respiratory distress syndrome. N Engl J Med. 2000;342(18):1301-8.
4. Griffiths MJD, McAuley DF, Perkins GD, Barrett N, Blackwood B, Boyle A, et al. Guidelines on the management of acute respiratory distress syndrome. BMJ Open Respir Res. 2019;6(1):e000420.
5. Fan E, Del Sorbo L, Goligher EC, Hodgson CL, Munshi L, Walkey AJ, et al. An Official American Thoracic Society/European Society of Intensive Care Medicine/Society of Critical Care Medicine Clinical Practice Guideline: Mechanical Ventilation in Adult Patients with Acute Respiratory Distress Syndrome. Am J Respir Crit Care Med. 2017;195(9):1253-63.
6. González SB, Menga G, Raimondi GA, Tighiouart H, Adrogué HJ, Madias NE. Secondary Response to Chronic Respiratory Acidosis in Humans: A Prospective Study. Kidney International Reports. 2018;3(5):1163-70.
7. Panitchote A, Mehkri O, Hastings A, Hanane T, Demirjian S, Torbic H, et al. Factors associated with acute kidney injury in acute respiratory distress syndrome. Ann Intensive Care. 2019;9(1):74.
8. Tignanelli CJ, Wiktor AJ, Vatsaas CJ, Sachdev G, Heung M, Park PK, et al. Outcomes of Acute Kidney Injury in Patients With Severe ARDS Due to Influenza A(H1N1) pdm09 Virus. Am J Crit Care. 2018;27(1):67-73.
9. Cerdá J, Tolwani AJ, Warnock DG. Critical care nephrology: management of acid–base disorders with CRRT. Kidney Int. 2012;82(1):9-18.

Supplemental Figure 1: Scheme of pCO2 adapted CKRT

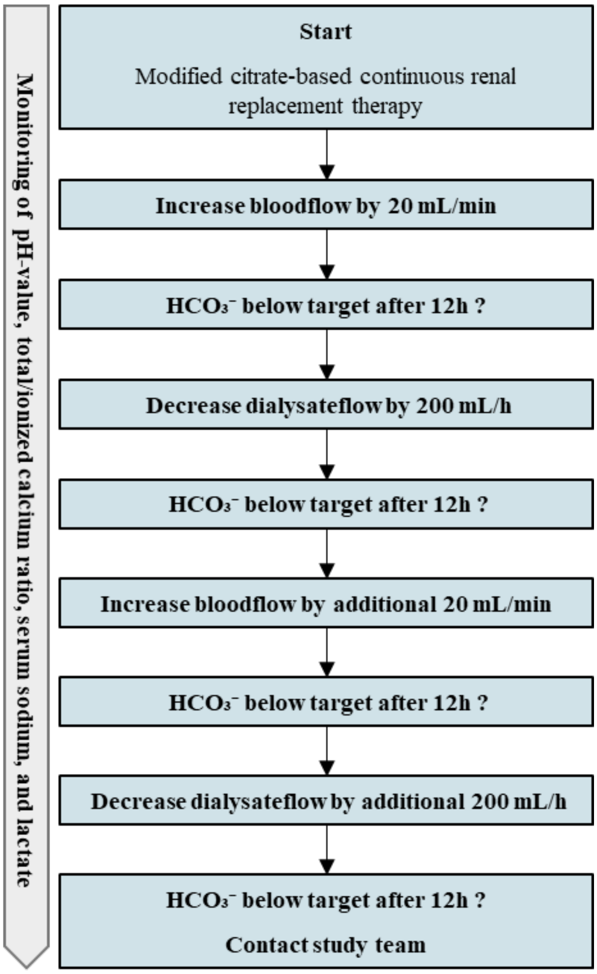

Supplemental Table 1: Amount of citrate added relative to blood-flow

**Blood-flow (ml/min)**	**Citate-flow per L blood-flow**	**Citrate-flow (mmol/h) (**Citrate solution flow (4 %sodium citrate, containing 136 mmol/L citrate ions)
100ml/min	4mmol/L	24 mmol/h citrate
120ml/min	4mmol/L	28,8 mmol/h citrate
140ml/min	4mmol/L	33,6 mmol/h citrate

Supplemental Table 2: Additional outcomes of the study participants

Characteristic	N[1]	Control<br>N = 16[2]	Intervention<br>N = 19[2]	Relative effect[3]<br>(CI)[4]	Odds[5]	p-value[6]
**Ventilation parameters:**						
**Horowitz index (PaO2/FiO2) [mmHg]**						
Day 1	33	173 (134, 194)	162 (133, 195)	0.49 (0.28 - 0.71)	0.98	0.96
Day 2	30	149 (130, 223)	167 (136, 219)	0.50 (0.26 - 0.73)	0.99	0.98
Day 3	26	178 (149, 220)	190 (131, 209)	0.46 (0.20 - 0.73)	0.86	0.77
Day 4	26	175 (142, 215)	187 (130, 198)	0.45 (0.19 - 0.72)	0.83	0.72
Day 5	26	166 (135, 239)	176 (128, 199)	0.50 (0.23 - 0.77)	0.99	0.98
Day 6	23	183 (133, 244)	165 (135, 200)	0.45 (0.17 - 0.73)	0.83	0.73
**Respiratory minute volume /kg PBW [L/min]**						
Day 1	34	145 (137, 177)	145 (134, 170)	0.45 (0.23 - 0.66)	0.80	0.61
Day 2	29	153 (137, 172)	149 (133, 177)	0.49 (0.26 - 0.72)	0.96	0.93
Day 3	26	174 (156, 214)	160 (132, 179)	0.32 (0.08 - 0.55)	0.47	0.12
Day 4	26	181 (166, 198)	147 (135, 189)	0.34 (0.11 - 0.56)	0.51	0.15
Day 5	26	178 (173, 195)	160 (131, 191)	0.29 (0.08 - 0.51)	0.42	0.06
Day 6	23	166 (153, 185)	163 (135, 184)	0.43 (0.17 - 0.69)	0.75	0.57
**Driving pressure [mBAR]**						
Day 1	35	14.00 (12.00, 17.25)	14.00 (12.50, 16.50)	0.48 (0.28 - 0.69)	0.93	0.86
Day 2	30	13.00 (12.00, 14.00)	14.00 (11.75, 16.00)	0.58 (0.36 - 0.80)	1.38	0.46
Day 3	26	13.50 (12.00, 14.75)	14.00 (11.00, 16.00)	0.46 (0.21 - 0.71)	0.86	0.75
Day 4	26	13.00 (11.25, 15.00)	12.50 (10.00, 16.00)	0.43 (0.12 - 0.67)	0.77	0.57
Day 5	26	13.00 (10.50, 14.75)	13.50 (10.00, 15.25)	0.49 (0.25 - 0.74)	0.98	0.96
Day 6	23	14.00 (12.00, 14.00)	13.50 (9.25, 15.00)	0.49 (0.23 - 0.76)	0.97	0.95
**Positive end-expiratory pressure [mBar]**						
Day 1	34	11.5 (10.0, 14.0)	16.0 (14.0, 17.0)	0.78 (0.61 - 0.95)	3.54	0.002
Day 2	29	12.00 (10.00, 15.00)	14.00 (12.75, 16.25)	0.67 (0.46 - 0.88)	2.06	0.10
Day 3	25	11.50 (10.25, 13.75)	15.00 (13.00, 16.50)	0.74 (0.52 - 0.96)	2.85	0.03
Day 4	25	10.50 (10.00, 13.75)	14.00 (12.50, 16.00)	0.70 (0.47 - 0.93)	2.30	0.09
Day 5	25	11.00 (9.00, 14.50)	14.00 (11.50, 15.50)	0.65 (0.40 - 0.90)	1.86	0.22
Day 6	22	13.0 (8.0, 15.0)	13.0 (11.0, 14.0)	0.53 (0.24 - 0.83)	1.15	0.81
**Inspiratory plateau pressure [mBar]**						
Day 1	35	27.0 (25.3, 27.0)	30.0 (27.5, 32.0)	0.76 (0.58 - 0.93)	3.14	0.005
Day 2	29	26.0 (24.0, 28.0)	28.0 (26.0, 31.3)	0.71 (0.52 – 0.91)	2.45	0.040
Day3	25	25.5 (24.3, 28.0)	28.0 (26.0, 31.5)	0.67 (0.44 - 0.90)	2.03	0.14
Day4	25	23.5 (22.0, 28.8)	27.0 (21.5, 30.0)	0.60 (0.36 - 0.84)	1.50	0.40
Day5	25	22.0 (21.3, 27.8)	28.0 (21.5, 30.0)	0.58 (0.34 - 0.83)	1.38	0.49
Day6	23	23.5 (22.0, 29.3)	26.0 (20.0, 29.0)	0.49 (0.22 - 0.76)	0.96	0.93
**Respiratory rate [breaths/min]**						
Day 1	34	22.4 (17.9, 23.7)	23.7 (19.8, 25.7)	0.60 (0.39- 0.80)	1.48	0.35
Day 2	29	18.5 (16.4, 23.8)	22.9 (20.5, 25.7)	0.64 (0.40 - 0.89)	1.81	0.23
Day 3	26	21.1 (18.4, 29.9)	22.9 (21.2, 24.8)	0.52 (0.22 - 0.82)	1.08	0.90
Day 4	26	21.2 (19.8, 23.7)	23.4 (21.5, 24.9)	0.62 (0.37 - 0.87)	1.62	0.33
Day 5	26	22.5 (20.1, 26.5)	20.7 (18.4, 22.5)	0.38 (0.09 - 0.66)	0.60	0.35
Day 6	23	23.9 (18.9, 25.1)	20.6 (19.6, 23.8)	0.44 (0.14 - 0.73)	0.77	0.65
**Other clinical endpoints:**						
Duration of stay in ICU in surviving patients [days]	11	35 (33, 59)	82 (53, 116)	0.83 (0.21 - 1.45)	5.00	0.17
Duration of mechanical ventilation in ICU-surviving patients [days]	11	34 (30, 56)	76 (49, 110)	0.83 (0.21 - 1.45)	5.00	0.17
Discharged from ICU	35	3 (19%)	8 (42%)			0.13
Free of CKRT at ICU discharge in ICU surviving patients	11	1 (33%)	3 (38%)			>0.99
**Norepinephrine dose (µg/kg/min)**						
Day 1	35	0.25 (0.12, 0.41)	0.13 (0.10, 0.19)	0.32 (0.11 - 0.52)	0.46	0.08
Day 2	30	0.12 (0.08, 0.31)	0.10 (0.05, 0.12)	0.38 (0.15 - 0.60)	0.60	0.26
Day 3	26	0.06 (0.04, 0.14)	0.09 (0.08, 0.10)	0.57 (0.30 - 0.85)	1.34	0.58
Day 4	26	0.08 (0.05, 0.13)	0.09 (0.06, 0.16)	0.55 (0.30 - 0.80)	1.21	0.69
Day 5	26	0.04 (0.00, 0.14)	0.10 (0.07, 0.18)	0.64 (0.39 - 0.90)	1.80	0.26
Day 6	23	0.12 (0.06, 0.19)	0.07 (0.03, 0.08)	0.25 (0.03 - 0.47)	0.34	0.03

Characteristic	N[1]	Control<br>N = 16[2]	Intervention<br>N = 19[2]	Relative effect[3]<br>(CI)[4]	Odds[5]	p-value[6]
**Bloodflow [ml/min]**						
Day 1	35	100 (100, 100)	120 (120, 120)	0.97 (0.92 - 1.03)	37.02	<0.001
Day 2	30	100 (100, 100)	120 (120, 120)	0.97 (0.90 - 1.04)	30.95	<0.001
Day 3	26	100 (100, 100)	120 (120, 120)	0.97 (0.90 - 1.04)	32.00	<0.001
Day 4	26	100 (100, 100)	120 (110, 120)	0.87 (0.74 - 0.99)	6.50	<0.001
Day 5	26	100 (100, 100)	120 (100, 120)	0.76 (0.60 - 0.92)	3.18	0.003
Day 6	23	100 (100, 100)	120 (120, 120)	0.85 (0.69 - 1.00)	5.50	<0.001
**Dialysatflow [ml/h]**						
Day 1	35	2,000 (2,000, 2,000)	2,000 (2,000, 2,000)	0.46 (0.29 - 0.62)	0.84	0.58
Day 2	30	2,000 (2,000, 2,150)	2,000 (1,950, 2,000)	0.37 (0.18 - 0.55)	0.58	0.14
Day 3	26	2,000 (2,000, 2,200)	2,000 (1,800, 2,100)	0.38 (0.16 - 0.59)	0.60	0.23
Day 4	26	2,000 (2,000, 2,200)	2,000 (1,900, 2,000)	0.37 (0.17 - 0.56)	0.58	0.17
Day 5	26	2,000 (2,000, 2,400)	2,000 (1,900, 2,000)	0.29 (0.11 - 0.48)	0.42	0.03
Day 6	23	2,100 (2,000, 2,400)	2,000 (2,000, 2,400)	0.44 (0.20 - 0.68)	0.78	0.59
**SOFA Score**						
Day 1	35	11.50 (10.75, 14.25)	14.00 (12.00, 14.50)	0.65 (0.44 - 0.86)	1.85	0.15
Day 2	30	12.00 (9.25, 14.00)	13.00 (12.00, 14.00)	0.59 (0.35 - 0.83)	1.45	0.43
Day 3	26	10.0 (7.5, 12.5)	13.0 (11.0, 14.5)	0.70 (0.46 - 0.94)	2.33	0.10
Day 4	26	10.0 (8.5, 12.0)	13.0 (11.5, 14.0)	0.74 (0.51 - 0.97)	2.79	0.05
Day 5	26	10.0 (8.5, 12.5)	13.0 (11.0, 14.5)	0.73 (0.52 - 0.95)	2.75	0.03
Day 6	23	11.00 (9.00, 12.75)	12.00 (10.00, 14.00)	0.64 (0.39 - 0.89)	1.80	0.25
**Serum sodium [mmol\L]**						
Day 1	34	143.85 (142.63, 144.73)	145.00 (143.88, 145.68)	0.70 (0.51 - 0.90)	2.39	0.04
Day 2	30	143.90 (143.00, 144.40)	146.15 (145.48, 147.65)	0.94 (0.84 - 1.05)	16.24	<0.001
Day 3	26	143.80 (142.65, 145.70)	146.50 (145.40, 148.00)	0.86 (0.71 - 1.01)	6.33	<0.001
Day 4	26	143.30 (142.00, 144.20)	145.20 (144.65, 145.95)	0.81 (0.60 - 1.02)	4.24	0.01
Day 5	26	143.20 (142.45, 144.90)	144.80 (143.75, 145.80)	0.67 (0.41 - 0.93)	2.06	0.18
Day 6	23	143.55 (142.18, 144.70)	144.90 (143.90, 145.40)	0.75 (0.53 - 0.97)	3.00	0.03
**Bilirubine [mg/dL]**						
Day 1	33	0.80 (0.30, 1.53)	1.15 (0.50, 3.03)	0.59 (0.38 - 0.80)	1.46	0.37
Day 2	30	0.60 (0.46, 0.90)	1.22 (0.58, 3.50)	0.70 (0.50 - 0.90)	2.37	0.05
Day 3	25	0.60 (0.34, 0.82)	1.75 (0.93, 3.96)	0.80 (0.61 - 0.99)	3.97	0.004
Day 4	26	0.70 (0.39, 1.17)	1.70 (0.75, 3.46)	0.75 (0.55 - 0.96)	3.07	0.02
Day 5	26	0.60 (0.42, 1.06)	2.02 (0.85, 3.42)	0.83 (0.66 – 1.00)	4.89	<0.001
Day 6	23	0.73 (0.49, 0.87)	1.48 (0.74, 2.84)	0.74 (0.52 - 0.96)	2.88	0.03
**Calciumdose [mmol/L]**						
Day 1	33	1.70 (1.50, 1.83)	1.70 (1.30, 1.90)	0.49 (0.27 - 0.70)	0.95	0.90
Day 2	30	1.50 (1.33, 1.70)	1.70 (1.50, 1.90)	0.63 (0.42 - 0.84)	1.70	0.22
Day 3	26	1.70 (1.50, 1.70)	1.70 (1.50, 1.90)	0.59 (0.40 - 0.83)	1.44	0.44
Day 4	26	1.50 (1.30, 1.70)	1.80 (1.50, 1.90)	0.65 (0.41 - 0.88)	1.84	0.20
Day 5	26	1.50 (1.20, 1.70)	1.80 (1.55, 1.90)	0.69 (0.47- 0.91)	2.27	0.08
Day 6	23	1.55 (1.30, 1.70)	1.70 (1.30, 1.90)	0.66 (0.40 - 0.93)	1.95	0.21
**Ionised serum calcium [mmol\L]**						
Day 1	33	1.20 (1.17, 1.24)	1.19 (1.13, 1.22)	0.39 (0.19 - 0.59)	0.64	0.28
Day 2	30	1.18 (1.15, 1.22)	1.16 (1.13, 1.17)	0.35 (0.12 - 0.57)	0.53	0.17
Day 3	26	1.16 (1.13, 1.17)	1.13 (1.10, 1.18)	0.43 (0.18 - 0.68)	0.76	0.59
Day 4	26	1.18 (1.13, 1.21)	1.18 (1.15, 1.21)	0.49 (0.24 - 0.74)	0.95	0.92
Day 5	26	1.17 (1.15, 1.20)	1.19 (1.17, 1.22)	0.61 (0.37 - 0.85)	1.54	0.37
Day 6	22	1.14 (1.13, 1.20)	1.16 (1.10, 1.21)	0.46 (0.19 - 0.72)	0.84	0.74
**Max. lactate level [mg/dL]**						
Day 1	32	15 (10, 26)	15 (10, 21)	0.46 (0.25 - 0.68)	0.86	0.73
Day 2	30	12 (10, 24)	13 (10, 19)	0.47 (0.24 - 0.70)	0.90	0.81
Day 3	26	12.0 (8.0, 15.5)	10.0 (9.0, 17.5)	0.50 (0.24 - 0.76)	0.99	0.98
Day 4	26	12.0 (8.5, 16.0)	12.0 (8.0, 14.5)	0.48 (0.22 - 0.73)	0.91	0.84
Day 5	26	10 (9, 15)	12 (9, 14)	0.53 (0.27 - 0.79)	1.12	0.83
Day 6	23	10.0 (8.0, 11.8)	11.0 (10.0, 14.0)	0.60 (0.34 - 0.87)	1.52	0.42

[1]Number of patients included in the analysis. The distribution per group can be found in Supplemental Table 5. [2]Median (IQR); n (%), [3]Relative effect p (Brunner-Munzel test), [4]Confidence interval, [5]Relative effect p/(1-p) [6]Continuous variable using the Brunner-Munzel Test and categorical variable using the Boschloo's Exact Test, PBW: predicted body weight; pCO2: Carbon dioxide partial pressure; ICU = intensive care unit, CKRT: continuous kidney replacement therapy

Supplemental Table 3: Individual bilirubin levels in mg/dL

Control group	Baseline	Day 1	Day 2	Day 3	Day 4	Day 5	Day 6
Patient 1	0.4	0.79	0.91	0.75	0.75	0.78	0.9
Patient 2	1.17	1.9	3				
Patient 3	0.33	0.21	0.29	0.34	0.35	0.44	0.49
Patient 4	0.2	0.29	0.5				
Patient 5	0.81	0.81	0.7	0.79	0.87	0.66	0.75
Patient 6	3.23	4.69	2.49	1.93	1.46	1.97	2.02
Patient 7	0.4	1.4					
Patient 8	2	2.13	1.75	1.8	1.69	1.73	1.86
Patient 9	0.31	0.3	0.28	0.25	0.27	0.26	0.23
Patient 10	0.29	0.19	0.74				
Patient 11	1.34	1.21	0.85	0.85	1.5	1.34	
Patient 12	0.18	0.19	0.46	0.38	0.38	0.58	0.77
Patient 13	2.4	2.9					
Patient 14	0.8	0.9	0.46	0.34	0.7	0.6	0.7
Patient 15	0.6	0.3	0.23	0.25	0.4	0.4	0.5
Patient 16	0.9	0.7	0.5	0.6	0.5	0.23	0.24
**Intervention group**	**Baseline**	**Day 1**	**Day 2**	**Day 3**	**Day 4**	**Day 5**	**Day 6**
Patient 1	4	4.98	6.18	6.19	6.34	6	
Patient 2	1	1.1	0.8	0.4	0.31	0.41	0.59
Patient 3	0.6	0.4	0.6	0.9	1.7	2.93	7.4
Patient 4	1.14	1.15	1.38				
Patient 5	0.46						
Patient 6	4	4.51	4.28	4.24	4.89	6.51	
Patient 7	1.05	0.9	0.99	1.1	0.87	0.8	0.53
Patient 8	0.24	0.24	0.28		1.89	1.41	2.84
Patient 9	1.17	1.17	1.06	1.01	1	0.91	0.77
Patient 10	3	3.03	3.29	3.12	2.88	3.37	2.63
Patient 11	0.18	0.15					
Patient 12	0.64	0.63	0.53	1.77	0.62	0.64	0.74
Patient 13	1.29	1.93	4.11	4.56	4.03	3.71	3.33
Patient 14	0.42	0.5	0.34	0.5	0.47	0.9	0.81
Patient 15	1.55						
Patient 16	3.93	3.93	2.3	2.3	2.05	2.02	1.48
Patient 17	0.4	0.16	0.4	0.3	0.3	0.7	0.3
Patient 18	1.6	1.56	1.79	1.73	1.66	2.4	2.21
Patient 19	5.7	4.4	4.16	4.5	4.08	3.46	3.38

Supplemental Table 4: 12-hour bicarbonate concentrations

Bicarbonate [mmol/L]	N	Control N = 16	Intervention N = 19
12 hours	35	27.20 (24.86, 28.30)	28.00 (27.10, 28.80)
24 hours	34	26.15 (25.00, 26.63)	29.01 (28.00, 30.25)
36 hours	31	26.70 (25.70, 27.50)	29.55 (29.03, 30.73)
48 hours	30	26.60 (25.38, 27.70)	30.55 (27.75, 31.20)
60 hours	26	25.80 (25.45, 27.10)	31.00 (29.45, 31.90)
72 hours	26	26.0 (25.4, 27.7)	30.8 (29.0, 31.9)
84 hours	26	27.4 (25.1, 29.0)	31.3 (27.2, 32.4)
96 hours	26	27.4 (25.9, 29.2)	30.9 (27.0, 31.5)
108 hours	26	27.10 (26.65, 29.30)	30.00 (28.10, 31.10)
120 hours	26	27.00 (25.60, 27.35)	29.40 (26.45, 30.80)
132 hours	23	27.10 (25.53, 28.63)	29.00 (28.40, 30.20)
144 hours	23	27.80 (27.30, 28.93)	29.00 (27.90, 30.70)

Values are median (IQR); N= number

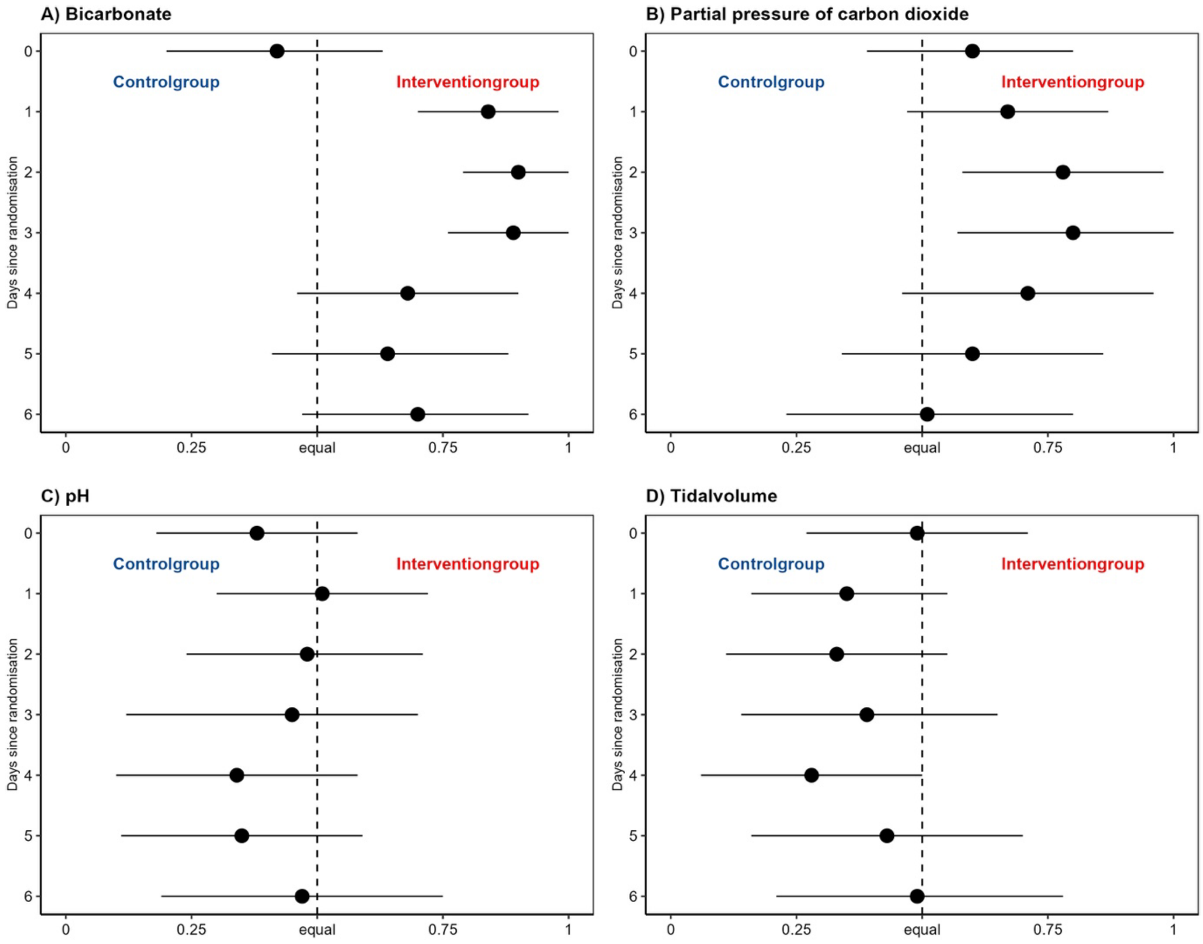

Supplemental Figure 2: Relative effects of the intervention on HCO_{3^-}, pCO2, pH and tidalvolume
A) Relative effect on bicarbonate concentration **B)** Relative effect on carbon dioxide partial pressure. **C)** Relative effect on pH. **D)** Relative effect on tidal volume.

Supplemental Table 5: Distribution into groups

**Included in analysis**	**N** (total)	**Control** N = 16 (45,7%)	**Intervention** N = 19 (54,3%)
Baseline	35	16 (100%)	19 (100%)
Day 1	34	16 (100%)	18 (95%)
Day 2	30	14 (88%)	16 (84%)
Day 3	26	11 (69%)	15 (79%)
Day 4	26	11 (69%)	15 (79%)
Day 5	26	11 (69%)	15 (79%)
Day 6	23	10 (63%)	13 (68%)

Values are numbers =N (%)
